# Efficient and versatile rapeseed transformation for new breeding technologies

**DOI:** 10.1111/tpj.70330

**Published:** 2025-07-10

**Authors:** Kea Ille, Siegbert Melzer

**Affiliations:** ^1^ Plant Developmental Biology and Physiology Kiel University Am Botanischen Garten 5 24118 Kiel Germany

**Keywords:** *Brassica napus*, recalcitrant winter rapeseed, transformation, WUSCHEL, CRISPR, *CLV3*, *SPL9*, *SPL15*

## Abstract

Many gene functions are widely studied and understood in Arabidopsis; however, the lack of efficient transformation systems often limits the application and verification of this knowledge in crop plants. *Brassica napus* L., a member of the Brassicaceae family, is usually transformed by *Agrobacterium*‐mediated hypocotyl transformation, but not all growth types are equally amenable to transformation. In particular, winter rapeseed, which requires vernalization to initiate flowering, is recalcitrant to *in vitro* regeneration and transformation. The analysis of gene functions in rapeseed is further complicated by the allotetraploid nature of its genome and the genome triplication within the *Brassica* genus, which has led to the presence of a large number of gene homologs for each Arabidopsis ortholog. We have established a transformation method that facilitates the regeneration of winter rapeseed by using the *WUSCHEL* gene from *Beta vulgaris*. This allowed us to efficiently transform a winter and spring rapeseed genotype in small‐scale experiments. As proof of principle, we targeted *BnCLV3* and *BnSPL9/15* with CRISPR/Cas9 and showed that entire gene families are effectively edited using this transformation protocol. This allowed us to simultaneously study many redundantly acting homologous genes in rapeseed. We observed mutant phenotypes for *BnCLV3* and *BnSPL9/15* in primary transformants, indicating that biallelic knockouts were obtained for up to eight genes. This allowed an initial phenotypic characterization to be performed already a few months after starting the experiment.

## Significance Statement

Winter rapeseed is recalcitrant to regeneration and transformation, and the complex *Brassica napus* genome complicates gene functional analyses. This study establishes an efficient small‐scale transformation method using the *Beta vulgaris WUSCHEL* gene, enabling rapid CRISPR/Cas9 editing of entire gene families in both winter and spring rapeseed. Up to eight genes were biallelically edited in primary transformants, allowing early phenotypic characterization.

## INTRODUCTION

Genetic variation is the driving force for establishing new traits in crop plants. New breeding techniques that employ gene editing are developing rapidly and offer many new methods to generate genetic variation. However, the transformation and regeneration of many crop plants is still a bottleneck for applying these techniques and for studying gene functions by molecular genetic approaches (Altpeter et al., 2016). This is even more challenging in polyploid crops that require efficient techniques to study highly redundant gene functions of many genes involved in a certain trait. Moreover, some crops or certain genotypes are considered recalcitrant to *Agrobacterium*‐mediated transformation, and recovering transgenic plants from these crops is difficult or impossible (Lee & Wang, 2023).

The improvement of crops, including the allotetraploid crop rapeseed (*Brassica napus* L.), with new breeding techniques is under constant development. As an important oilseed crop, rapeseed is cultivated worldwide and is adapted to distinct environments by different life history traits. Spring rapeseed can be sown and harvested within the same year, whereas semi‐winter and winter types are biennial and require a mild or strict cold period (vernalization), respectively. *B. napus* is closely related to the model plant Arabidopsis; however, transformation and genetic engineering are not as straightforward as in Arabidopsis (Clough & Bent, 1988). Some studies have shown that *Agrobacterium*‐mediated transformation of *B. napus* through floral dipping is possible (Ding et al., 2019; Li et al., 2010; Ren et al., 2012; Tan et al., 2011; Verma et al., 2008; Wang et al., 2003); however, it is not an easy procedure to implement routinely. Consequently, most transgenic rapeseed plants have been generated through tissue‐culture approaches.

In tissue culture, the tissue type plays an important role in the success of the transformation, and various tissues have been used, including haploid microspore‐derived embryos (Boutilier et al., 2002), epicotyls, and higher stem segments (Cao Chu et al., 2020). However, most studies have used either cotyledons (Bhalla & Singh, 2008; Moloney et al., 1989) or hypocotyls (Cardoza & Stewart, 2003; De Block et al., 1989; Radke et al., 1988) for transgenic studies. In addition to culture conditions, medium composition and the concentration of phytohormones, *Agrobacterium* strains and densities are critical for transformation success. But even if rapeseed is generally amenable to transformation, protocols are often still genotype dependent, and not all relevant genotypes can be successfully transformed. The spring cultivar Westar is mostly used, whereas other studies also report the transformation of different spring rapeseed and some semi‐winter rapeseed. Recent reports about transgenic winter rapeseed are lacking, and although some early studies have demonstrated transformation of winter rapeseed (Chandler et al., 2005; Damgaard et al., 1997; De Block et al., 1989), it appears to be mostly recalcitrant to transformation. However, to be able to study winter rapeseed–specific traits such as vernalization requirement, it is still important to generate mutants. Therefore, improving the transformation and regeneration of winter rapeseed, in particular, is crucial.

Recently, the efficiency of regeneration and transformation of other recalcitrant crop species or genotypes could be improved by the expression of morphogenic genes. The transformation of wheat has been improved by using wheat *GROWTH‐REGULATING FACTOR 4* (*GRF4*) and its cofactor *GRF‐INTERACTING FACTOR 1* (*GIF1*) (Debernardi et al., 2020). Moreover, the overexpression of *GRF5* enhanced the transformation rates of recalcitrant sugar beet varieties, soybean, sunflower, and maize (Kong et al., 2020). Likewise, the transformation efficiency in wheat, triticale, rye, barley, and maize was enhanced by *WUSCHEL‐RELATED HOMEOBOX 5* (*WOX5*) (Wang et al., 2022), which belongs to the same homeobox gene family as *WUSCHEL* (*WUS*). *WUS* has been widely used to improve the transformation and regeneration of various plants such as poplar (Pan et al., 2022), cotton (Bouchabké‐Coussa et al., 2013), or coffee (Arroyo‐Herrera et al., 2008). Together with the gene *BABYBOOM* (*BBM*), *ZmWUS2* enabled the transformation of previously non‐transformable maize inbred lines and other crops including sorghum (Lowe et al., 2016). Also, *BBM* alone helped to overcome the bottleneck of regeneration and transformation in recalcitrant sweet pepper varieties (Heidmann et al., 2011). However, to avoid pleiotropic effects, which often occur when *WUS* and *BBM* are overexpressed, an excision or inducible expression system (Heidmann et al., 2011; Lowe et al., 2018) was necessary. To avoid pleiotropic effects, Hoerster et al. (2020) used the cell non‐autonomous nature of WUS proteins and developed an altruistic transformation strategy for maize in which the ZmWUS2 protein initiates somatic embryogenesis of neighboring cells transformed with a gene of interest without *ZmWUS2* integration.

However, the use of morphogenic genes for regeneration and transformation improvement in rapeseed is limited. Overexpression of *BnBBM* in rapeseed induced somatic embryo formation on shoot tissues (Boutilier et al., 2002), whereas overexpression of *B. napus SHOOTMERISTEMLESS* (*STM*) enhanced the yield of microspore‐derived embryos (Elhiti et al., 2010). Kong et al. (2020) demonstrated that overexpression of *AtGRF5* and *BnGRF5‐LIKE* increased transgenic callus formation but did not promote transgenic shoot development. To date, no transformation protocols employing morphogenic genes have been developed to improve rapeseed transformation, despite the absence of transgenic winter rapeseed.

Here, we present the use of the *WUS* ortholog from *Beta vulgaris* in *Agrobacterium*‐mediated rapeseed hypocotyl transformation of *B. napus*. The overexpression of *BvWUS* facilitated the regeneration of transgenic winter rapeseed Express617 plants and enabled us to develop an easy and rapid protocol suitable for small‐scale applications.

Furthermore, we observed CRISPR/Cas9 biallelic editing of multiple gene copies with redundant functions already in primary Express617 and Westar transformants, which allows the early analysis of gene functions.

## RESULTS

### *BvWUS* facilitates shoot regeneration in winter rapeseed Express617

The overexpression of plant developmental regulators has facilitated and improved the regeneration of different recalcitrant plant species. We initially aimed for a simple and efficient transformation protocol for sugar beet, which is known to be recalcitrant to *Agrobacterium*‐mediated transformation. We tested whether the regeneration capacity and the transformation rate could be improved by expressing the Arabidopsis *WUS* gene and its putative ortholog from sugar beet. For this, we cloned the respective cDNAs into the binary vector p9o‐LH‐35s‐ocs (DNA Cloning Service, Hamburg) under the control of the CaMV 35S promoter (35S::*AtWUS* and 35S::*BvWUS*) and used these constructs for *Agrobacterium*‐mediated transformation. However, neither the overexpression of *AtWUS* nor *BvWUS* in different sugar beet tissues led to the recovery of transgenic sugar beet plants.

**Figure 1:**
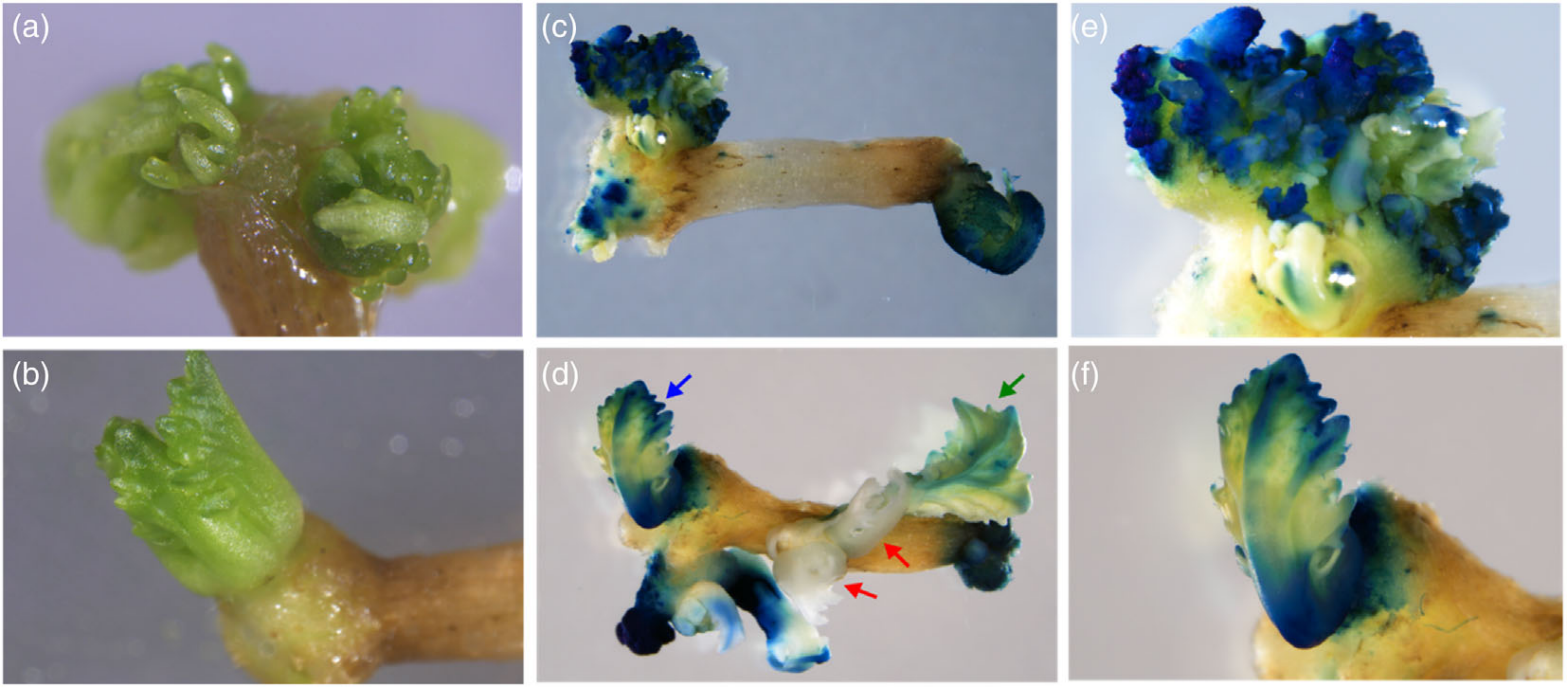
Express617 hypocotyl on selection medium after transformation with *AtWUS* or *BvWUS* and *GUS*. (a) 35S::*AtWUS*. Overproliferation of somatic embryos on hypocotyl ends without proper shoot formation. (b) 35S::*BvWUS*. Distinct single shoots grew from the callus at the hypocotyl end. (c) 35S::*AtWUS* and 35S::*GUS*. Blue‐stained and white somatic embryos on hypocotyl ends after GUS staining. (d) 35S::*BvWUS* and 35S::*GUS*. Blue‐stained shoots on hypocotyl after GUS staining (blue arrow). Destained green shoots appear yellowish (green arrow), whereas white shoots bleached out due to kanamycin selection (red arrows). (e) Close‐up of (c). (f) GUS‐stained shoot from (d).

In parallel, we were developing a regeneration and transformation strategy for the recalcitrant winter rapeseed Express617, which we could not regenerate or transform in our lab before. Over the course of multiple years, 6612 hypocotyl segments were transformed with a 35S::*GUS* construct; however, no transgenic shoots were recovered (Table 1). Therefore, we tested whether the expression of either *AtWUS* or *BvWUS* under the control of the CaMV 35S promoter would improve the regeneration and transformation efficiency in Express617 and give rise to transgenic plants. Accordingly, we carried out a small‐scale hypocotyl transformation with the 35S::*AtWUS* and 35S::*BvWUS* constructs using 295 and 282 hypocotyl segments, respectively. Under kanamycin selection, the transformed hypocotyl segments initially formed callus at the cut ends. After 3 weeks, the hypocotyl segments transformed with the 35S::*AtWUS* construct showed massive embryogenic growth on the callus. Numerous somatic embryos formed and, over time, more embryogenic structures were produced, and the callus swelled substantially (Figure 1a). None of these embryos fully developed into shoots as we did not induce embryo germination.

**Table 1:** Recovered transgenic plants from different experiments

Transgene 1 (TG1)	Transgene 2 (TG2)	Genotype	No. of explants	No. of experiments	No. of plants with only TG1	No. of plants with only TG2	No. of plants with TG1 + TG2	Total	KO
	35S::*GUS*	Express617	6612	22	—	0	—	0	—
35S::*AtWUS*		Express617	295	1	0	—	—	0	—
35S::*BvWUS*		Express617	282	1	10	—	—	10	—
35S::*AtWUS*	35S::*GUS*	Express617	411	1	0	0	1 chimera		—
35S::*BvWUS*	35S::*GUS*	Express617	424	1	30	5	—	54	—
35S::*BvWUS*	pDGE652‐*CLV3*	Express617	430	1	9	2	4	15	4
35S::*BvWUS*	pDGE652‐*CLV3*	Westar	312	1	9	2	2	13	3
35S::*BvWUS*	pDGE652‐*SPL9*/*15*	Westar	345	1	1	1	6	8	2

*Note*: KO: number of plants with a full knockout phenotype. –: not applicable.

By contrast, hypocotyls transformed with the 35S::*BvWUS* construct developed many individual and distinct shoots directly from calli at the hypocotyl ends after 6 weeks (Figure 1b). In total, 115 shoots regenerated, most of which bleached out under kanamycin selection, immediately following the co‐cultivation phase. Nevertheless, 10 shoots stayed green, developed roots on rooting medium, and contained the *BvWUS* transgene. The plants expressing *BvWUS* did not show aberrant phenotypes and looked like the WT, indicating that mutant phenotypes could potentially be observed even in the presence of the transgene. This demonstrates that expression of the distantly related *BvWUS* (Figure S1) in winter rapeseed hypocotyls improves regeneration and transformation efficiency.

### Co‐transformation with *BvWUS* can lead to transgenic plants that only contain the gene of interest

On the basis of the observation that most of the regenerated 35S::*BvWUS* shoots bleached out under kanamycin selection, we reasoned that due to the cell non‐autonomous activity of WUS proteins (Daum et al., 2014), WUS might have moved from transgenic cells into neighboring cells lacking the transgene. There, WUS proteins initiated shoot regeneration, and thus, many shoots regenerated that did not contain the transgene and the selection marker. On the basis of this assumption, we tested whether employing a co‐transformation strategy with 35S::*AtWUS* and 35S::*BvWUS* together with another construct containing a gene of interest would facilitate the regeneration of plants not only harboring the *WUS* transgene and the gene of interest, but also of plants only containing the gene of interest. A high proportion of regenerated plants containing only the gene of interest would later eliminate the need to identify segregants without the *WUS* transgene.

We mixed *Agrobacterium* containing the binary plasmid pBin19::pSH4 (35S::*GUS*) (Holtorf et al., 1995) and *Agrobacterium* with 35S::*AtWUS* or 35S::*BvWUS* and carried out a co‐transformation of Express617 hypocotyls. Similar to the transformation experiment with *WUS* only, hypocotyls co‐transformed with 35S::*GUS* and 35S::*AtWUS* showed many somatic embryos (Figure 1c,e), and hypocotyls co‐transformed with 35S::*GUS* and 35S::*BvWUS* showed *de novo* shoot formation (Figure 1d,f). To determine the integration of the 35S::*GUS* transgene, we GUS‐stained the hypocotyls with regenerated tissues.

**Figure 2:**
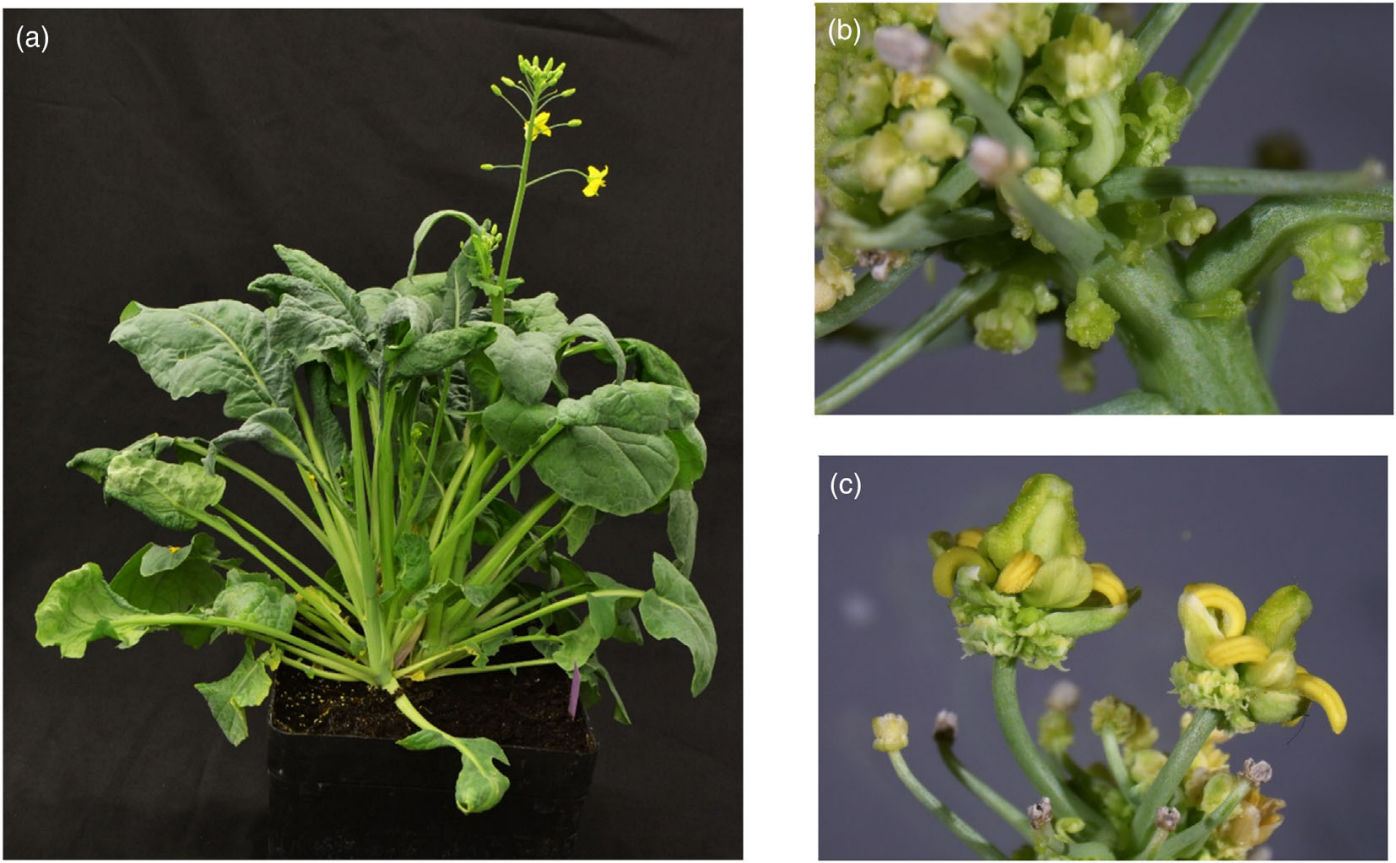
Chimeric Express617 plant expressing *AtWUS*. (a) Chimeric 35S::*AtWUS* plant at flowering stage. (b) Abnormal tissue proliferation along the inflorescence of the chimeric part of the 35S::*AtWUS* plant. (c) Misshapen flowers of a chimeric 35S::*AtWUS* T_0_ plant.

Hypocotyls co‐transformed with 35S::*GUS* and 35S::*AtWUS* showed numerous GUS‐stained embryos and some areas of developing callus and embryos without any GUS staining (Figure 1c,e). From this experiment, one plant was recovered, which produced multiple shoots (Figure 2a), of which one had crinkled leaves and showed abnormal tissue proliferation along the inflorescence (Figure 2b), whereas other shoots developed normally. This indicates that only that particular shoot contained the 35S::*AtWUS* transgene, while the remaining shoots had a WT phenotype, implying that the regenerated plant was chimeric. Flowers on the 35S::*AtWUS* shoot were misshapen with degenerated floral organs and consequently did not produce any seeds (Figure 2c). As the expression of *AtWUS* only rarely facilitated the recovery of shoots and had detrimental effects in the recovered chimeric plant, such as the overproliferation of tissues and infertility, expressing *AtWUS* was not suitable for improving the transformation and regeneration of rapeseed.

We found that hypocotyls co‐transformed with 35S::*BvWUS* and 35S::*GUS* mostly led to the recovery of co‐transformed plants that contained both transgenes, and plants that only contained the 35S::*BvWUS* transgene (Table 1, Figure 1d,f). However, a small number of plants with only the gene of interest were recovered, although these plants were underrepresented among the regenerated transgenic plants.

### Co‐transformation with *BvWUS* and a CRISPR vector led to efficient editing of multiple *BnCLV3* alleles

**Figure 3:**
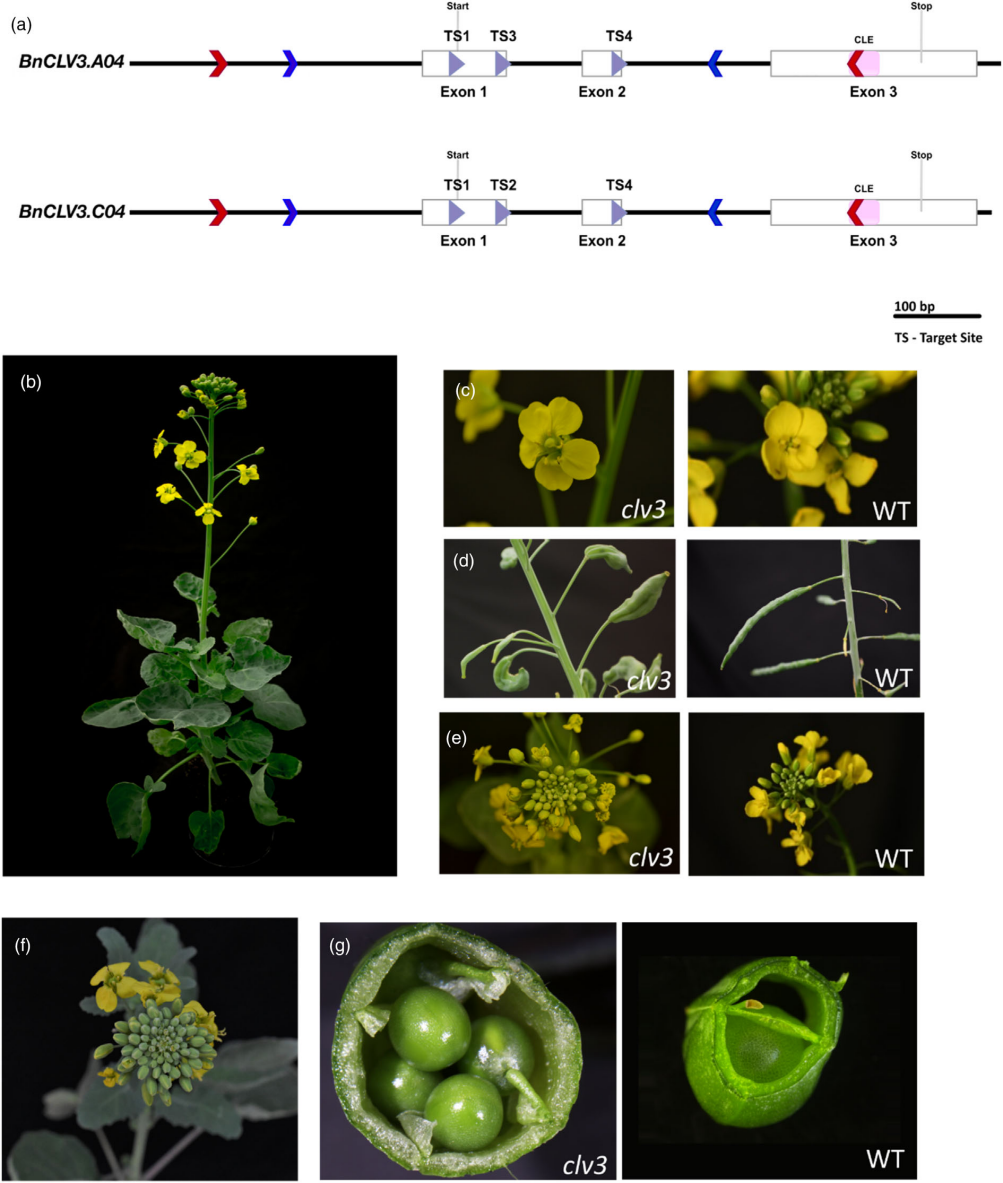
*BnCLV3* editing in Westar. (a) Gene structure of *BnCLV3.A04* and *BnCLV3.C04*. Blue arrows indicate the position of conserved primers with barcodes for amplicon sequencing. Red arrows show the position of homolog‐specific primers, and purple arrows show the position of target sites. The translational start and stop sites are indicated as well as the CLE domain. (b) T_0_ Westar plant 7 with *clv3* loss‐of‐function phenotype. (c) Flower of *clv3‐W7* with five petals and enlarged gynoecium and a WT flower. (d) Multilocular silique of *clv3‐W7* and WT siliques. (e) Raceme of *clv3‐W7* and the WT. (f)–(g) *clv3* loss‐of‐function phenotype in progeny of *clv3‐W7* (T_1_). (f) Topview of the raceme. (g) Multilocular silique alongside a WT silique.

Next, we aimed to establish a co‐transformation strategy with 35S::*BvWUS* and CRISPR/Cas9 for gene editing. As proof of principle, we chose to knock out both *BnCLAVATA3* (*BnCLV3*) homologs (*BnCLV3.A04* and *BnCLV3.C04*) in the winter rapeseed Express617 and spring rapeseed Westar. It was previously demonstrated that *clv3* loss‐of‐function mutants show a clear phenotype with increased leaf number, an enlarged SAM, and multilocular siliques (Yang et al., 2018), which fulfilled our requirement for a phenotype that was easy to detect. We targeted both *BnCLV3* genes with four sgRNAs (Figure 3a) cloned in the plant transformation vector pDGE652 (zCas9i cloning kit, Stuttmann et al., 2021). Target sites were positioned in exons 1 and 2 near and after the start codon to generate frameshift mutations or larger deletions between target sites. The target sites TS1 and TS4 were conserved between *BnCLV3.A04* and *BnCLV3.C04*. The target site TS2 on *BnCLV3.C04* and the target site TS3 on *BnCLV3.A04* differed by one SNP 7 bp upstream of the PAM site (Table S1). This resulted in three different target sites per allele and thus a total of 12 target sites in all four *BnCLV3* alleles.

We carried out a small‐scale 35S::*BvWUS* co‐transformation with 430 and 312 hypocotyl segments of Express617 and Westar, respectively. The transformation of Express617 gave rise to six plants that contained the CRISPR T‐DNA, of which four were double transformants. Moreover, we obtained four Westar plants that had the CRISPR T‐DNA integrated, of which two were double transformants (Table 1). Already in the T_0_ generation, we observed four and three plants with a *clv3* loss‐of‐function phenotype in Express617 and Westar, respectively. T_0_ *clv3* mutants showed an increased number of leaves and petals, as well as enlarged gynoecia and multilocular siliques (Figure 3b–e). The presence of a loss‐of‐function phenotype in a high proportion of T_0_ plants indicated efficient editing in all four alleles of *BnCLV3* in both Express617 and Westar.

### Analysis of *BnCLV3* gene‐editing events

**Figure 4:**
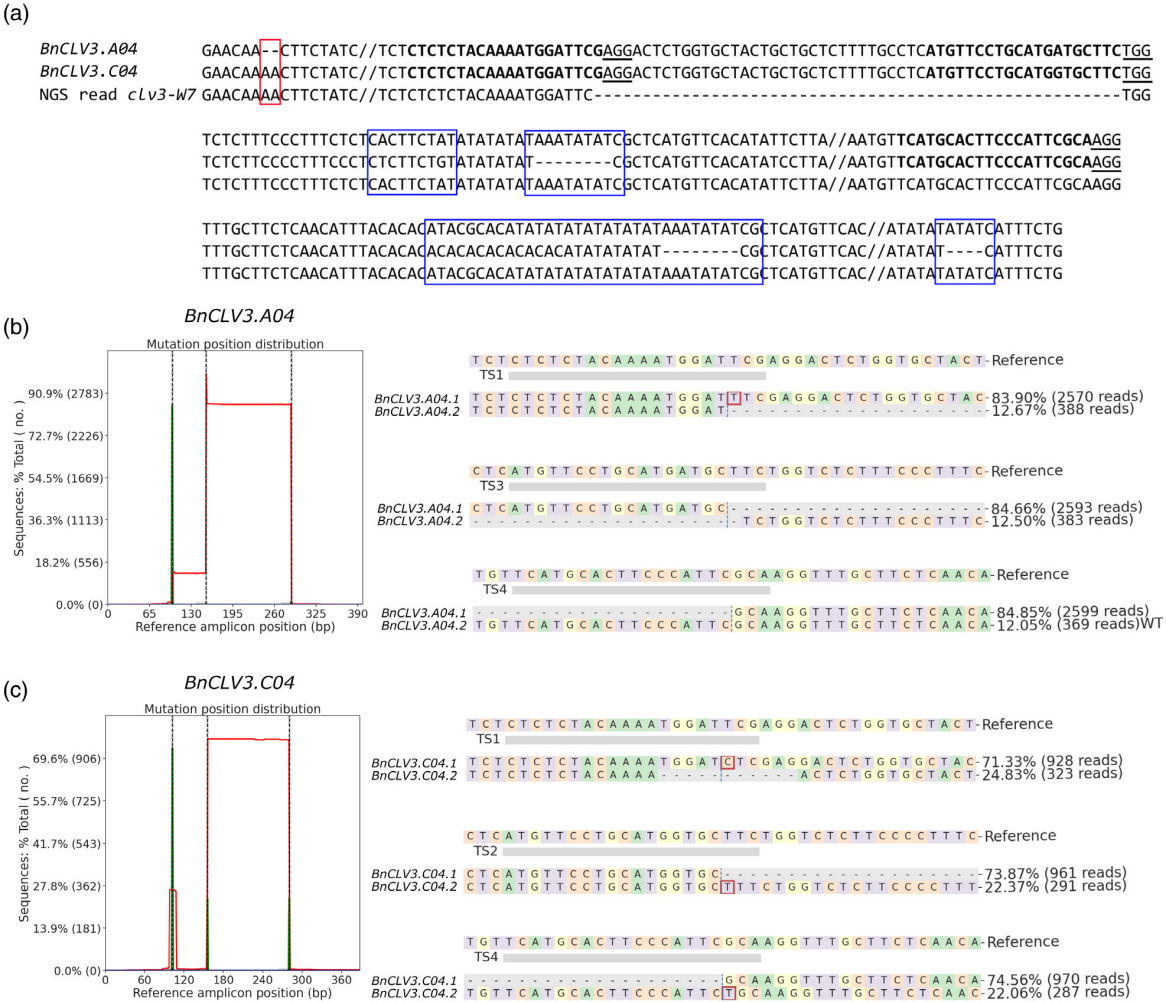
Analysis of gene editing in T_0_ plants by amplicon sequencing. (a) PCR recombination in one exemplary NGS read of *clv3‐W7*. The red rectangle represents the affiliation of the InDel to the homolog on C04 and the blue rectangles to SNP and InDel of the homolog on A04. Gene‐editing events in mutant *clv3‐W7* in (b) *BnCLV3.A04* and (c) *BnCLV3.C04*. Left: CRISPResso2 output showing the frequency of insertions (green) and deletions (red) across the entire amplicon, considering only modifications that overlap with the quantification window in *BnCLV3* genes. The Y‐axis depicts the number of reads that were mapped to the reference amplicon. The X‐axis shows the positions on the PCR amplicon in bp, and the dotted lines show the predicted CRISPR/Cas9 cleavage site 3 bp downstream of the PAM site on the amplicon. Sharp peaks indicate that small insertions or deletions occurred. Wider peaks indicate the occurrence of larger insertions or deletions. Right: Visualization of the distribution of identified alleles around the cleavage site in *BnCLV3* genes for all target sites. Nucleotides are indicated by unique colors (A = green; C = red; G = yellow; T = purple). Red rectangles highlight inserted sequences. Horizontal dashed lines indicate deleted sequences. The vertical dashed line indicates the predicted cleavage site.

We analyzed the gene‐editing events of T_0_ plants by amplicon sequencing (NGS AmpliconEZ, Azenta, Leipzig, Germany). Amplicon sequencing requires PCR fragments of up to 500 bp spanning the target sites; however, designing gene‐specific primers of homologous genes in such a small nucleotide range in rapeseed is challenging due to its allotetraploid nature and consequent sequence similarities. Thus, we designed conserved primers to amplify both *BnCLV3* copies simultaneously, including regions with SNPs to distinguish between the two copies (Figure 3a). We analyzed demultiplexed reads with CRISPResso 2.0 (Clement et al., 2019) in CRISPRessoPooled mixed mode and further validated results by checking the output bam files in Integrative Genome Viewer (Robinson et al., 2011). However, we observed a high proportion of PCR recombination, which can occur if very similar genes from gene families or polyploid organisms such as rapeseed are amplified simultaneously (Meyerhans et al., 1990). If the amplification of a template stops prematurely, the incompletely transcribed strand can serve as a primer for the other homolog in our case, which leads to chimeric PCR products. We frequently observed that SNPs from both *BnCLV3* gene copies were included within one read (Figure 4a). These chimeric PCR products prevented us from determining to which homologs the mutations belonged. To circumvent these difficulties, we developed a strategy to minimize PCR recombination. Therefore, we designed homolog‐specific primers around the target sites for an initial PCR and subsequently conducted a nested PCR with barcode primers (Figure 3a), which minimized PCR recombination.

In Westar plant 7 (*clv3‐W7*), 11 out of 12 target sites were edited, resulting in a full knockout and a loss‐of‐function *clv3* phenotype in T_0_. We identified biallelic editing in both genes, resulting in two different loss‐of‐function alleles per gene. Collectively, four target sites showed an insertion of 1 bp, one showed a deletion of 11 bp, and one maintained the WT sequence. We further observed large deletions of 54 bp, 125 bp, and 133 bp between target sites (Figure 4b,c).

**Figure 5:**
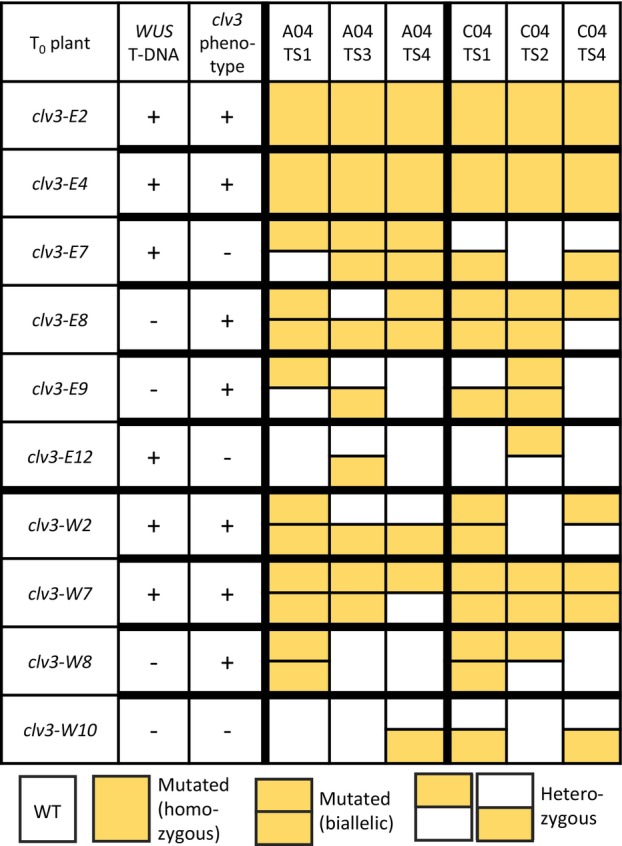
Genotyping of primary Westar and Express617 transformants from co‐transformation with 35S::*BvWUS* and pDGE652_*CLV3*. Integration of *BvWUS* T‐DNA was determined by PCR. The *clv3* loss‐of‐function phenotype is indicated by a +. Gene editing at each target site was analyzed by amplicon sequencing.

Out of 16 analyzed alleles of four transgenic Westar plants, 14 were loss‐of‐function alleles, and among six transgenic Express617 plants, 21 out of 24 analyzed alleles were loss‐of‐function alleles. As indicated by the *clv3* phenotype of seven T_0_ plants, all alleles and most target sites were edited in these plants. In three Westar and four Express617 *clv3* mutants, sequencing results confirmed the loss‐of‐function phenotype and showed gene editing in all four alleles leading to a full knockout (Figure 5, Table S2). Overall, the number of target sites and edited alleles, and the frequent occurrence of large deletions between target sites showed highly efficient gene editing in our experiments.

To obtain T‐DNA‐free T_1_ Westar mutants and to check for the inheritance of the mutations and phenotype, we self‐pollinated T_0_ Westar mutants and grew T_1_ plants. Plants were screened for the *BvWUS* and CRISPR T‐DNA by PCR and, in the case of the CRISPR transgene, on the basis of red fluorescence of the FAST marker encoded within the T‐DNA. Therefore, we germinated seeds on wet filter paper and screened seedlings for red fluorescence (Figure S2). Among 48 T_1_ progeny of *clv3‐W2*, we identified no plants that lacked either T‐DNA, suggesting the presence of multiple integrations. Because *clv3‐W2* was edited biallelically in both genes, all progeny also showed the *clv3* phenotype (Figure 3f,g). This was also the case for the progeny of *clv3‐W7* and *clv3‐W8*, in which we determined the integration of one and no *BvWUS* transgene, respectively, and more than three CRISPR T‐DNAs for *clv3‐W7* and two CRISPR T‐DNAs for *clv3‐W8*. The segregation pattern of the progeny of *clv3‐W10* indicated the integration of only one CRISPR T‐DNA. Because the mutations in T_0_ were only heterozygous in certain target sites, the *clv3* phenotype was absent and the progeny segregated for the *clv3* loss‐of‐function phenotype (Table S3).

For Express617, we selected T_1_ seedlings on the basis of red fluorescence of the FAST marker encoded within the T‐DNA. T_1_ seedlings without red fluorescence and thus without the CRISPR T‐DNA were grown to check for the *clv3* phenotype. All progeny of *clv3‐E2*, *clv3‐E4*, *clv3‐E8*, and *clv3‐E9* showed the *clv3* loss‐of‐function phenotype, whereas the progeny of *clv3‐E7* and *clv3‐E12* segregated for the phenotype.

In summary, all mutations and the *clv3* loss‐of‐function phenotype were transmitted to the T_1_ generation and T‐DNA‐free plants could be identified.

### Eight *BnSPL9/15* genes can be efficiently edited simultaneously in Westar

**Figure 6:**
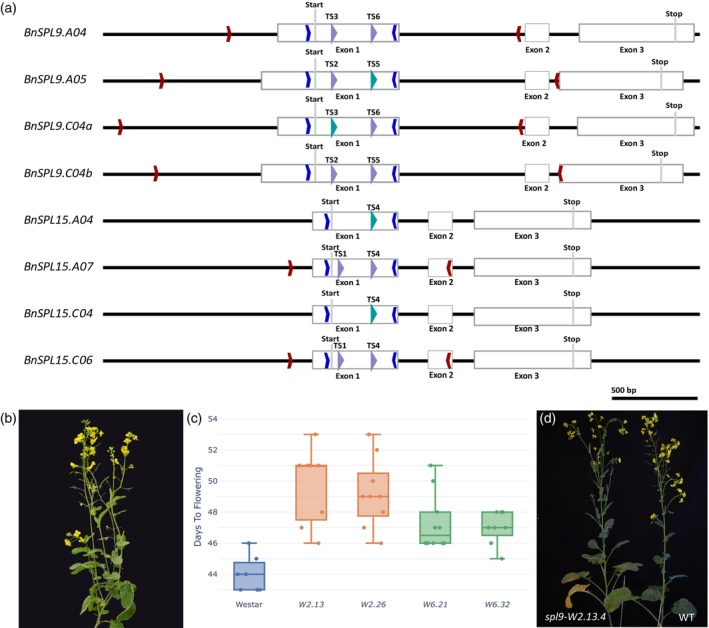
*BnSPL9/BnSPL15* editing in Westar. (a) Gene structure of *BnSPL9* genes and *BnSPL15* genes. Blue arrows indicate the position of conserved primers with barcodes for amplicon sequencing. Red arrows show the position of homolog‐specific primers, and purple arrows show the position of target sites. Turquoise arrows show the position of target sites with mismatches. The translational start and stop sites are indicated. (b) T_0_ *spl9/15‐W2* with reduced apical dominance. (c) T_2_ progeny flowered later than Westar WT. (d) Exemplary T_2_ mutant *spl9/15‐W2.13.4* with reduced apical dominance flowered later than the Westar WT.

Because we obtained high proportions of *clv3* knockout phenotypes in T_0_ plants, and observed that most target sites were edited, we aimed to knock out a greater number of genes simultaneously in spring rapeseed Westar. Therefore, we chose to target *BnSPL9* and *BnSPL15*, which both have four homologs in rapeseed, with six different sgRNAs. We designed target sites in the eight genes *BnSPL9.A04, BnSPL9.A05, BnSPL9.C04a, BnSPL9.C04b, BnSPL15.A04, BnSPL15.A07, BnSPL15.C04*, and *BnSPL15.C06*, targeting each gene once or twice in the first exon after the start codon to generate frameshift mutations or larger deletions between target sites (Figure 6a). TS1 is conserved between the genes *BnSPL15.A07* and *BnSPL15.C06*, and TS2 between the genes *BnSPL9.A05* and *BnSPL9.C04b*. TS3 from *BnSPL9.A04* has one mismatch on *BnSPL9.C04a* at position 18 upstream of the PAM site. TS4 is conserved between *BnSPL15.A07* and *BnSPL15.C06* and additionally has two mismatches at positions 19 and 20 upstream of the PAM site of *BnSPL15.A04* and one mismatch at position 20 of *BnSPL15.C04*. TS5 from *BnSPL9.C04b* has one mismatch at position 12 on *BnSPL9.A05*, and TS6 is conserved between *BnSPL9.A04* and *BnSPL9.C04a* (Table S1). In total, 14 sites in all 8 genes or 28 sites in 16 alleles were targeted.

After the co‐transformation of 345 Westar hypocotyl segments, we obtained eight transgenic plants, of which seven contained the CRISPR T‐DNA. Furthermore, five of these plants also contained the 35S::*BvWUS* transgene and were thus co‐transformants (Table 1). T_0_ mutants *spl9/15‐W2* and *spl9/15‐W8* showed a reduction in apical dominance (Figure 6b) as described for the *spl9 spl15* Arabidopsis mutant (Schwarz et al., 2008) suggesting a full knockout of all eight genes.

We sequenced *BnSPL9.C04a* and *BnSPL9.C04b* in plants *spl9/15‐W1*, *spl9/15‐W2*, *spl9/15‐W4*, and *spl9/15‐W6* and found large deletions between target sites TS2 and TS5 in *BnSPL9.C04b* in all four plants (Figure S3). We further observed editing of TS3, which had one mismatch on *BnSPL9.C04a* in all four analyzed plants. Notably, both alleles of *BnSPL9.C04a* in plant *spl9/15‐W2* were only edited at the target site with mismatches but not at the conserved target site TS6 (Figure S4). We analyzed one plant with a loss of apical dominance phenotype (*spl9/15‐W2*) and one WT phenotype (*spl9/15‐W6*) in more detail and sequenced all targeted genes of these plants. In *spl9/15‐W2*, all genes were edited in both alleles including *BnSPL15.A04* and *BnSPL15.C04*, which only had targets with mismatches leading to a full knockout of all genes. Out of 28 target sites in 16 alleles, 21 were edited, whereas seven remained WT. By contrast, in T_0_ plant *spl9/15‐W6*, only TS1 on one allele of *BnSPL15.A07* and TS4 on one allele of *BnSPL15.A04* were not edited. We found mutations in 26 out of 28 target sites; however, due to the lack of editing in one allele of *BnSPL15.A04*, this gene was not knocked out. In addition, TS5 in *BnSPL9.A05* with a mismatch at position 12 was edited in both alleles (Table S4).

We grew T_1_ plants of both T_0_ plants *spl9/15‐W2* and *spl9/15‐W6*, identified mutants that lacked both T‐DNAs, and sequenced the selected plants (Table S5). We grew the T_2_ generation from two T_1_ plants per T_0_ plant (*spl9/15‐W2.13*, *spl9/15‐W2.26*, *spl9/15‐W6.21*, and *spl9/15‐W6.32*), in which all eight genes were knocked out (Table S5). Ten T_2_ plants per line and the Westar WT control grew at 20°C and 16 h light in a growth chamber. We recorded the opening of the first flower and the plant habitus. The flowering time of mutant lines ranged between 45 and 51 days after sowing, meaning that all mutant lines flowered later than the WT (Figure 6c). All lines showed a loss of apical dominance (Figure 6d).

### Introduction of the 35S:: *BvWUS* cassette into a CRISPR vector

Most of the regenerated plants from co‐transformation experiments contained the *BvWUS* T‐DNA. The co‐transformation strategy is necessary if, for example, overexpression constructs should be transformed as the 35S::*GUS* construct. However, integration of the 35S::*BvWUS* cassette into a CRISPR vector would ease the selection of transgene‐free progeny in later generations. Hence, a new CRISPR vector was constructed that included the entire 35S::*BvWUS* cassette. As the pCas9‐TPC vector had provided reliable editing events from co‐transformation with 35S::*BvWUS* in Express617 (unpublished data), it was used as a base. The PcUbi4‐2 promoter driving the *Cas9* gene was exchanged for a CaMV 35S promoter, which is better suited for transformation in tissue culture, because it has high activity in the plant callus (Stefanov et al., 1994). Furthermore, the *bar* gene was excised and an *NPTII* gene driven by a nos promoter was introduced. To enable editing of multiple genes with several sgRNAs, a Golden Gate cloning cassette with the *ccdB* gene was introduced. For testing the vector, the previously used *BnCLV3* sgRNAs were introduced into the new vector by Golden Gate cloning (Figure S6). With this vector, a small hypocotyl transformation of Express617 was carried out. Three transgenic plants with the integrated T‐DNA were recovered (Table S7). All three plants were edited; however, only one plant had the *clv3* loss‐of‐function phenotype. *clv3‐E2‐pCAS9* was edited in eight of the 12 target sites and had the *clv3* loss‐of‐function phenotype. In *clv3‐E1‐pCAS9*, all four alleles were edited, but the plant lacked the *clv3* loss‐of‐function phenotype. In two alleles at TS1, the start codon ATG was deleted. A second in‐frame ATG, 42 bp downstream of the first start codon is present in *BnCLV3*, which might facilitate the production of a shortened protein from which the active CLE domain at the C‐terminal end can still be processed. Because the two other target sites were not edited in this mutant, the *clv3* loss‐of‐function phenotype was absent. This also occurred in the third recovered plant *clv3‐E3‐pCAS9*. Overall, the CRISPR vector with the integrated 35S::*BvWUS* cassette effectively initiated shoot regeneration and enabled the recovery of plants with biallelic mutations. Combining the 35S::*BvWUS* cassette with CRISPR components in one vector will ease the removal of the transgene in subsequent generations.

Collectively, our experiments showed that using the distantly related *WUS* gene from sugar beet does not lead to overproliferation of embryonic tissues in transgenic plants. Instead, it leads to the formation of organized shoots that are indistinguishable from those of WT plants. Moreover, a co‐transformation strategy with *BvWUS* demonstrated that many genes and target sites can be effectively edited simultaneously, in this case, up to eight genes and 26 target sites in 16 alleles.

## DISCUSSION

The genetic analysis of growth‐type‐specific traits has been difficult in all growth types of allotetraploid rapeseed because a reliable protocol for winter rapeseed transformation was lacking. Most studies that produced transgenic winter rapeseed were performed around 30 years ago and were not reproduced in more recent studies, despite seemingly straightforward transformation protocols (Damgaard et al., 1997; De Block et al., 1989).

*BnSVP* was edited and analyzed in semi‐winter rapeseed (Ahmar et al., 2022), which does not have a strict vernalization requirement; however, studying *SVP* in winter rapeseed would be more informative regarding the vernalization response.

We have developed a co‐transformation strategy for recalcitrant winter rapeseed Express617 with the morphogenic gene *BvWUS*. Using 35S::*BvWUS* has enabled the transformation and regeneration of winter rapeseed Express617, and the hypocotyl transformation allowed us the easy, rapid, and small‐scale generation of CRISPR/Cas9 Express617 winter and Westar spring rapeseed mutants. Using this protocol, the function of multiple gene homologs and whole gene families in Express617 and Westar can now be analyzed. This protocol could potentially also improve the regeneration and transformation of other recalcitrant rapeseed genotypes.

Our co‐transformation strategy is based on simple *Agrobacterium*‐mediated hypocotyl transformation (De Block et al., 1989). Hypocotyls are easily accessible and simple to handle in comparison with other tissues. The maintenance of the tissue cultures is undemanding due to the minimal necessary steps because the medium is changed and shoots are excised every 3 weeks. Moreover, the amount of starting material needed is low, and in our experiments, up to 430 hypocotyl segments (equivalent to approximately 90 seedlings) were sufficient to generate several transgenic Express617 and Westar plants and CRISPR full knockouts in the T_0_ generation. LaManna et al. (2022) reported using 520 seedlings for *Agrobacterium*‐mediated hypocotyl transformation of spring Westar and recovered only four plants that carried the T‐DNA. Furthermore, to obtain T_1_ seeds, the whole procedure took about 12 months. With our co‐transformation protocol, T_1_ Express617 and Westar transgenic seeds can be obtained with minimal labor within eight and six months, respectively (Figure S5).

The overexpression of genes such as *GRF*, the chimera *GRF*–*GIF*, and *WOX5* improved the transformation of recalcitrant sugar beet (Kong et al., 2020) and wheat (Debernardi et al., 2020; Wang et al., 2022), respectively. Using these genes was advantageous because they did not lead to any abnormal phenotypes; however, the use of *BnGRF5* in rapeseed increased the amount of transgenic callus but not the number of transgenic plants (Kong et al., 2020).

Another commonly used morphogenic gene is *WUS*, but simply overexpressing *WUS* can lead to severe pleiotropic effects. Overexpression of the endogenous *WUS* gene in Arabidopsis under the control of the 35S promoter led to abnormal phenotypes in seedlings and impaired development (Zuo et al., 2002). A 17‐β‐estradiol‐inducible system helped to regulate *WUS* expression by restricting it to a certain time period, which enabled the formation of somatic embryos only during a short time window and led to the normal development of regenerated plants. The overexpression of *WUS* from different species apart from Arabidopsis has shown similar effects. Overexpression of the endogenous *BrWUSa* in turnip also resulted in sterile and abnormal plants, which was also overcome by an estradiol‐inducible system (Liu et al., 2022). The same system also helped to regenerate transgenic white spruce (Klimaszewska et al., 2010). Another strategy to limit *WUS* expression is the excision of the *WUS* cassette. Combining *ZmBBM* and *ZmWUS2* without their removal before regeneration also led to aberrant phenotypes and sterile plants in maize (Lowe et al., 2016). Furthermore, the ectopic expression of *AtWUS* in species such as cotton also caused abnormal somatic embryos (Zheng et al., 2014).

In our case, the expression of *AtWUS* and *BvWUS* in winter rapeseed showed remarkable differences. As previously described for other plant species, the ectopic expression of *AtWUS* in rapeseed also led to an aberrant phenotype and sterility. Whole plants could only be recovered when they were chimeric, suggesting that the constitutive expression of *AtWUS* hindered the development of fertile plants. However, instead of using elaborate induction or excision systems to limit *AtWUS* expression in rapeseed hypocotyls, expressing the distantly related *BvWUS* (Figure S1) was sufficient to directly induce shoot formation instead of somatic embryos and the regeneration of transgenic plants.

In our previous attempts to transform Express617 without 35S::*BvWUS*, we hardly regenerated any shoots from transformed hypocotyls and no transgenic plants (Table 1). Using the 35S::*BvWUS* construct, we initially observed that many shoots formed on the callus, but most bleached out under kanamycin selection. We reasoned that the non‐transgenic shoots formed due to the movement of cell non‐autonomous BvWUS proteins from cells carrying the *BvWUS* T‐DNA to non‐transgenic cells. Similarly, the movement of ZmWUS2 has been exploited for altruistic transformation of maize, and the transient expression of *ZmWUS2* and the simultaneous transformation of another T‐DNA enabled the regeneration of transgenic plants that lacked the *ZmWUS2* construct (Hoerster et al., 2020).

On this basis, we developed a co‐transformation strategy in which we mixed two *Agrobacterium* strains: one containing the 35S::*BvWUS* expression cassette and the other containing a construct of interest, for example, GUS or CRISPR. Throughout all experiments, only a small proportion of regenerated transgenic plants contained solely the construct of interest and not the 35S::*BvWUS* cassette (between 17 and 50%). In *B. napus*, ZS6 co‐transformation efficiencies of up to 81% were obtained when two *Agrobacterium* strains, each containing a different selection marker, were mixed in a ratio of 1:1 (Liu et al., 2020). In our case, most transgenic plants contained the 35S::*BvWUS* cassette with co‐transformation efficiencies between 14 and 62.5%, indicating that the regeneration of shoots is more efficient when the 35S::*BvWUS* cassette is integrated. In the altruistic transformation of maize, the co‐transformation efficiency was low due to the strong expression of genes encoding WUS2 and CRC transcription factors in plants containing the altruistic T‐DNA and the subsequent inhibition of growth of these plants (Hoerster et al., 2020). We reason that the low similarity between the BvWUS and the endogenous BnWUS proteins (Figure S1a) enables the regeneration of phenotypically normal rapeseed plants and prevents the overproliferation of somatic embryos and aberrant phenotypes that are observed when using the more similar AtWUS. BvWUS and AtWUS contain the conserved homeobox and WUS‐box (Figure S1b); however, they are variable in the rest of the protein sequence. Whereas the homology of the AtWUS protein to the BnWUS proteins might be high enough to recognize target genes and thus induce somatic embryogenesis, the homology of BvWUS might be too low for interaction with the target genes in rapeseed. It would be interesting to further test whether a refinement of *BvWUS* expression would be beneficial for regeneration or if stronger expression would lead to similar overproliferation as with *AtWUS*.

Due to its allotetraploidy, the *B. napus* genome contains multiple copies per gene. It is therefore necessary to target and knock out several genes simultaneously to study gene functions. Several studies showed that this is not always trivial. In semi‐winter rapeseed J9712 and J9707, two gene copies of *BnIDA* and *BnTT8*, respectively, were targeted by CRISPR/Cas9 (Wu et al., 2022; Zhai et al., 2020). In J9712, only seven out of the 30 transgenic plants exhibited editing events and none of the plants were knocked out for both targeted *BnIDA* genes in the T_0_ generation. Zhai et al. (2020) observed five plants with seeds that had a yellow seed coat and thus a full knockout of both *BnTT8* genes in T_0_; however, out of 333 transgenic plants, only 48 plants were edited, and only five for all alleles of these two genes. The same semi‐winter line was previously used for editing two *CLV3* genes (Yang et al., 2018). 494 T_0_ plants carrying the T‐DNA were recovered and only 51 of these plants were edited, and eight had a knockout phenotype. Also several studies that used CRISPR/Cas9 in Westar only obtained loss‐of‐function mutants in the T_1_ or T_2_ generation (LaManna et al., 2022; Tu et al., 2024).

In our experiments targeting *CLV3* in Westar and Express617, we recovered four and six plants that carried the CRISPR T‐DNA, respectively. All plants with the CRISPR T‐DNA were edited, and many plants were loss‐of‐function mutants displaying the described *clv3* phenotype (4/6 Express617 and 3/4 Westar plants). We were further able to increase the number of genes that were edited simultaneously, as shown in our CRISPR experiment targeting *BnSPL9* and *BnSPL15* in Westar. Again, we identified gene editing in all six plants carrying the CRISPR T‐DNA. In two plants, all eight targeted *BnSPL9* and *BnSPL15* genes were edited in both alleles, which resulted in a full knockout in T_0_. We often observed large deletions between two or three target sites in both CRISPR experiments, and no obvious differences between editing of different target sites. Moreover, our selected targets with mismatches were also efficiently edited. Collectively, these results indicate an exceptionally high efficiency of gene editing in our experiments.

Che et al. (2022) also observed that CRISPR/Cas9 gene editing was more efficient when they used *ZmWUS2* in the altruistic transformation of sorghum. They hypothesized that WUS activity might alter chromatin accessibility and thus might enable more efficient gene editing in open chromatin regions, which might also be a reasonable explanation for our high editing efficiencies when using *BvWUS*.

Another reason or additional factor for efficient editing might be the choice of the CRISPR vector. The pDGE vectors (Stuttmann et al., 2021) were specifically designed for multiplex editing of genes and have an intronized *Cas9* gene, which improved editing efficiencies (Grützner et al., 2021). Stuttmann et al. (2021) regenerated transgenic *Nicotiana benthamiana* plants from *Agrobacterium*‐mediated transformation that were mutated in all eight targeted genes, mostly biallelically, with an overrepresentation of homozygous mutations. This is also consistent with our observation in rapeseed where an unexpectedly high number of T_0_ plants displayed full knockout phenotypes.

For ease of use and to circumvent the removal of two transgenes for CRISPR/Cas9, we integrated the 35S::*BvWUS* cassette into a modified pCAS9‐TPC vector. Apart from exchanging the promoter of the *Cas9* gene, we additionally integrated a kanamycin selection marker and a Golden Gate cassette. The small experiments that targeted *CLV3* in Express617 showed that transgenic plants with loss‐of‐function alleles for all genes and a *clv3* phenotype can be obtained using this vector.

In summary, we have developed an efficient transformation and gene‐editing strategy for *B. napus* and used it to obtain the first gene‐edited Express617 winter rapeseed. Winter rapeseed Express617 has been used in numerous studies in recent years (Calderwood et al., 2021; Matar et al., 2021; Schilbert et al., 2023) and has an available reference genome (Lee et al., 2020). The distantly related *WUS* gene from sugar beet enabled the regeneration of transgenic winter rapeseed by directly inducing shoots and avoided the laborious construction of inducible systems for the temporally limited expression of *AtWUS*.

## MATERIALS AND METHODS

### Plant material and transformation

*Brassica napus* winter inbred line Express617 and spring rapeseed Westar were used for all transformation experiments. Hypocotyl transformation was conducted based on De Block et al. (1989) with modifications. Seeds were sterilized for 5 min in 70% EtOH, followed by 20 min in NaOCl with 6% active chlorine, and washed with sterile water. Subsequently, seeds were germinated on germination medium (4.9 g L^−1^ MS salts incl. vitamins and 500 mg L^−1^ MES (M0255, Duchefa, Haarlem, Netherland), 30 g L^−1^ sucrose, 7 g L^−1^ phyto agar, pH 5.7) and grown in low light for 1 week. *Agrobacterium tumefaciens* strain GV3101::pMP90 harboring the binary plasmids was grown overnight in LB medium with the appropriate antibiotics (rifampicin, gentamycin, spectinomycin) at 28°C. Bacteria were pelleted and resuspended in wash buffer supplemented with 10 μm acetosyringone (2.45 g L^−1^ MS salts incl. vitamins (Duchefa M0222), 500 mg L^−1^ MES, 0.01% (v/v) Silwet L‐77, pH 5.7) and the OD_600_ was adjusted to 0.1. For co‐transformation, *Agrobacterium* culture harboring the 35S::*BvWUS* vector was mixed in a ratio of 1:1 with *Agrobacterium* culture containing the vector with the gene of interest, whereas for the co‐transformations with the binary vector 35S::*AtWUS*, a ratio of 1 (35S::*AtWUS*): 9 (35S::*GUS*) was used. Hypocotyls were cut into about 1 cm long segments, submerged in *Agrobacterium* solution, and incubated for 15 min. Before placing the hypocotyl segments on co‐cultivation medium (modified after Cardoza & Stewart, (2003): 4.9 g L^−1^ MS salts incl. vitamins and 500 mg L^−1^ MES (Duchefa M0255), 30 g L^−1^ sucrose, 3 g L^−1^ Gelrite, 1 mg L^−1^ 2,4‐D, 10 μm acetosyringone), they were dabbed on an empty Petri dish to remove excess *Agrobacterium* solution. Hypocotyls were co‐cultivated with *Agrobacterium* for 48 h in the dark. Afterwards, hypocotyls were washed in wash buffer supplemented with 400 mg L^−1^ Timentin. Hypocotyls were then placed on regeneration medium (4.9 g L^−1^ MS salts incl. vitamins and 500 mg L^−1^ MES (Duchefa M0255), 20 g L^−1^ sucrose, 7 g L^−1^ phyto agar, 2 mg L^−1^ BAP, 0.01 mg L^−1^ Picloram, 0.01 mg L^−1^ NAA, 5 mg L^−1^ AgNO_3_, 400 mg L^−1^ Timentin, 100 mg L^−1^ kanamycin, pH 5.7) and transferred to fresh medium every 3 weeks. Westar hypocotyls were transferred to regeneration medium with reduced BAP concentration (0.5 mg L^−1^ BAP) after 2 weeks. Hypocotyls were cultured in a tissue‐culture room with 16 h light at 20–24°C until shoots appeared. Shoots were then excised and transferred to rooting medium (4.9 g L^−1^ MS salts incl. vitamins and 500 mg L^−1^ MES (Duchefa M0255), 20 g L^−1^ sucrose, 3 g L^−1^ Gelrite, 0.2 mg L^−1^ NAA, 0.2 mg L^−1^ IBA, 5 mg L^−1^ AgNO_3_, 400 mg L^−1^ Timentin, 100 mg L^−1^ kanamycin, pH 5.7). When the roots were grown sufficiently, rooted plants were transferred to soil and grown in a climate chamber (16 h light, 20°C). Express617 plants were vernalized at 4°C for 8 weeks (16 h light).

### Overexpression vectors

To construct the overexpression vectors, floral buds from Arabidopsis Col‐0 and *B. vulgaris* were isolated and RNA was extracted with the Plant RNA Kit, peqGOLD (VWR, Darmstadt, Germany). The RNA was treated with DNase I and reverse‐transcribed into cDNA with the First Strand cDNA Synthesis Kit (Thermo Fisher Scientific, Darmstadt, Germany). Primers with *Bam*HI and *Eco*RI restriction sites were used to amplify the ORF of the *AtWUS* (AT2G17950) and *BvWUS* genes by PCR with Phusion™ High‐Fidelity DNA Polymerase. The *BvWUS* gene was identified by a BLAST search in the reference genome RefBeet1.2 (Dohm et al., 2014) with *AtWUS* as a query. Fragments were blunt‐end cloned into pJET1.2 (CloneJET PCR Cloning Kit; Thermo Fisher Scientific), and sequences were validated by Sanger sequencing (IKMB, Institute for Clinical Molecular Biology, Kiel, Germany). pJET1.2 containing the correct sequences and the overexpression vector B425‐p9o‐LH‐35s‐ocs (DNA Cloning Service, Hamburg, Germany) were then digested with the respective restriction enzymes, and ORFs were ligated into the overexpression vector. Vectors were verified by restriction digestion, to generate the vectors B425‐*AtWUS* and B425‐*BvWUS*. Both vectors were transformed into *A*. *tumefaciens* strain GV3101::pMP90.

### zCas9i cloning

The zCas9i cloning kit (Stuttmann et al., 2021) was used to assemble the CRISPR vectors. The genes *BnCLV3, BnSPL9*, and *BnSPL15* were identified by BLAST searching the Arabidopsis CDS against the reference genomes of Express617 (Lee et al., 2020) and Westar (Song et al., 2020). The respective ID of the CDS can be found in Table S9. The target sites were designed with the help of CRISPR‐P 2.0 (Liu et al., 2017) and checked against the reference genomes of Express617 and Westar. Target sites and oligonucleotides are listed in Table S1 and were ordered from Eurofins Genomics. Oligonucleotides were annealed and ligated into shuttle vectors, and sgRNAs were assembled into pDGE652 as described by Stuttmann et al. (2021). Plasmids were validated by restriction digestion and Sanger sequencing.

### pCas9‐TPC

The pCas9‐TPC binary vector (Fauser et al., 2014) was modified for tissue culture by exchanging the Pc::Ubi10 promoter driving the *Cas9* gene for a CaMV 35S promoter, which is known to be active in early tissue‐culture stages. All PCRs were carried out with Q5 High‐Fidelity DNA Polymerase (NEB), and primers can be found in Table S6. The CaMV 35S promoter was amplified from the gateway destination vector pB7WG2D (Karimi et al., 2002), *Eco*RI restriction sites were added to the amplicon by PCR, and amplicons were subcloned into pJET1.2. The PcUbi4‐2 promoter driving the *Cas9* gene was cut out by *Eco*RI restriction from the pCas9‐TPC vector, and the 35S promoter was ligated into these restriction sites. In addition, the *bar* gene for Basta resistance was exchanged for a kanamycin resistance gene (*NEOMYCIN PHOSPHOTRANSFERASE II, NPTII*) for easier selection in tissue culture. Therefore, the nos::*NPTII* cassette from the pK7WG2D vector (Karimi et al., 2002) was amplified with primers with attached *Hin*dIII restriction sites, subcloned into pJET1.2, and after verification of the sequence, ligated into *Hin*dIII sites of the pCas9‐TPC vector. The vector was further modified for the use of Golden Gate cloning of sgRNAs and for the integration of the 35S::*BvWUS* cassette. To enable Golden Gate cloning via *Bsa*I sites, two already existing *Bsa*I sites were removed with Q5 Site‐Directed Mutagenesis (NEB) according to the manual. The *ccd*B cassette for Golden Gate cloning of multiple sgRNAs was amplified from pDGE652 (Grützner et al., 2021), the *Xba*I restriction sites were attached, and the fragment was ligated into *Spe*I sites of the vector. Lastly, the 35S::*BvWUS* cassette was amplified from the vector B425‐*BvWUS*, *Xba*I sites were attached, and then ligated into *Avr*II restriction sites, resulting in the vector pCas9‐WUS. All PCRs were carried out with Phusion High‐Fidelity DNA Polymerase, and the PCR fragments were blunt‐end subcloned into pJET1.2 and validated by restriction digestion and Sanger sequencing. For ligating subcloned fragments from pJET1.2 vectors into end vectors, restriction digestion of both vectors was carried out using the respective restriction enzymes. The final vector was verified by restriction digestion and was completely Sanger sequenced. To integrate sgRNAs, shuttle vectors from the zCas9i cloning kit were used, and Golden Gate cloning was carried out according to the instructions for the zCas9i cloning kit (Stuttmann et al., 2021).

### GUS staining

Co‐transformation was carried out with GV3101::pMP90 harboring the binary plasmid pBin19::pSH4 (Holtorf et al., 1995) with the *uid*A gene under the control of the CaMV 35S promoter. For the GUS staining, hypocotyls or leaf samples were submerged in GUS substrate solution (1× PBS buffer, 30 μl X‐Gluc solution (0.03 g X‐Gluc in 300 μl DMSO), 2 μl Triton X100) and incubated at 37°C overnight. The tissue was destained with 70% ethanol.

### Genotyping

DNA was isolated after a modified protocol from Dellaporta et al. (1983). To confirm the presence of the integrated transgenes, PCR was conducted with primers binding within the *BvWUS* transgene or in the *nptII* gene of the CRISPR vectors. CRISPR‐positive plants were further checked for editing in the respective genes. All PCRs for amplicon sequencing were carried out with Q5 polymerase (NEB, Frankfurt, Germany). Gene‐specific primers spanning the target sites were used to generate gene‐specific amplicons. With these amplicons as templates, a nested PCR was conducted using primers that carried barcodes, which facilitated the pooling of samples. Up to five plants per sample were pooled and amplicons were sequenced by Amplicon EZ (Genewiz, Leipzig, Germany). For the analysis of pooled sequences, paired‐end reads were merged with fastp (Chen, 2023) with standard settings for paired‐end reads and the ‐‐dont_eval_duplication parameter. Reads were then demultiplexed by using a *grep* command strategy in Unix. Demultiplexed reads were used for CRISPResso2 analysis (Clement et al., 2019). CRISPRessoPooled was run in mixed mode with previously merged single‐end reads with the following parameters and settings: ‐‐min_reads_to_use_region 5 ‐‐demultiplex_only_at_amplicons ‐‐bam_output ‐‐min_frequency_alleles_around_cut_to_plot 2 ‐‐allele_plot_pcts_only_for_assigned_reference ‐‐default_min_aln_score 0 –flexiguide_seq –flexiguide_homology 80. Alignments were manually checked in IGV (Robinson et al., 2011).

## AUTHOR CONTRIBUTIONS

KI carried out the experiments. KI and SM contributed to experimental design, data analysis, and manuscript writing.

## CONFLICT OF INTEREST

We have no conflicts of interest to disclose.

## Supporting information


**Figure S1.** Evolutionary relationships of taxa.
**Figure S2.** *clv3* T_1_ seedlings exhibiting red fluorescence from the FAST marker.
**Figure S3.** CRISPResso2 results for *BnSPL9.C04b* in T_0_ plants.
**Figure S4.** CRISPResso2 results for *BnSPL9.C04a* in T_0_ plants.
**Figure S5.** Overview and timeline of the *BvWUS* co‐transformation procedure.
**Figure S6.** T‐DNA of the CRISPR vector pCas9‐WUS.

**Table S1.** Target sites for *BnCLV3* and *BnSPL9/15*.
**Table S2.** Gene editing in T_0_ Westar and Express617 *clv3* mutants.
**Table S3.** Integration of transgenes in T_1_ progeny.
**Table S4.** Gene editing in two T_0_ Westar *spl9/15* mutants.
**Table S5.** Gene editing in T_1_ Westar progeny of *spl9/15‐W2* and *spl9/15‐W6*.
**Table S6.** Primers used for the construction of the vector pCas9‐WUS.
**Table S7.** Gene editing in T_0_ *clv3* Express617 plants transformed with the pCAS9‐WUS vector.
**Table S8.** Primers used in this study.
**Table S9.** Gene information.

## Data Availability

There are no data used in the manuscript, which have to be stored in repositories.
