# Supplementary material for: Efficient and versatile rapeseed transformation for new breeding technologies
Source: Plant J. 2025 Jul 10;123(1):e70330. doi: 10.1111/tpj.70330 (PMC12245476; doi:10.1111/tpj.70330)
Supplement: Supplementary file 1 — Figure S1. Evolutionary relationships of taxa. Figure S2. clv3 T1 seedlings exhibiting red fluorescence from the FAST marker. Figure S3. CRISPResso2 results for BnSPL9.C04b in T0 plants. Figure S4. CRISPResso2 results for BnSPL9.C04a in T0 plants. Figure S5. Overview and timeline of the BvWUS co‐transformation procedure. Figure S6. T‐DNA of the CRISPR vector pCas9‐WUS. [file TPJ-123-0-s002.pdf]

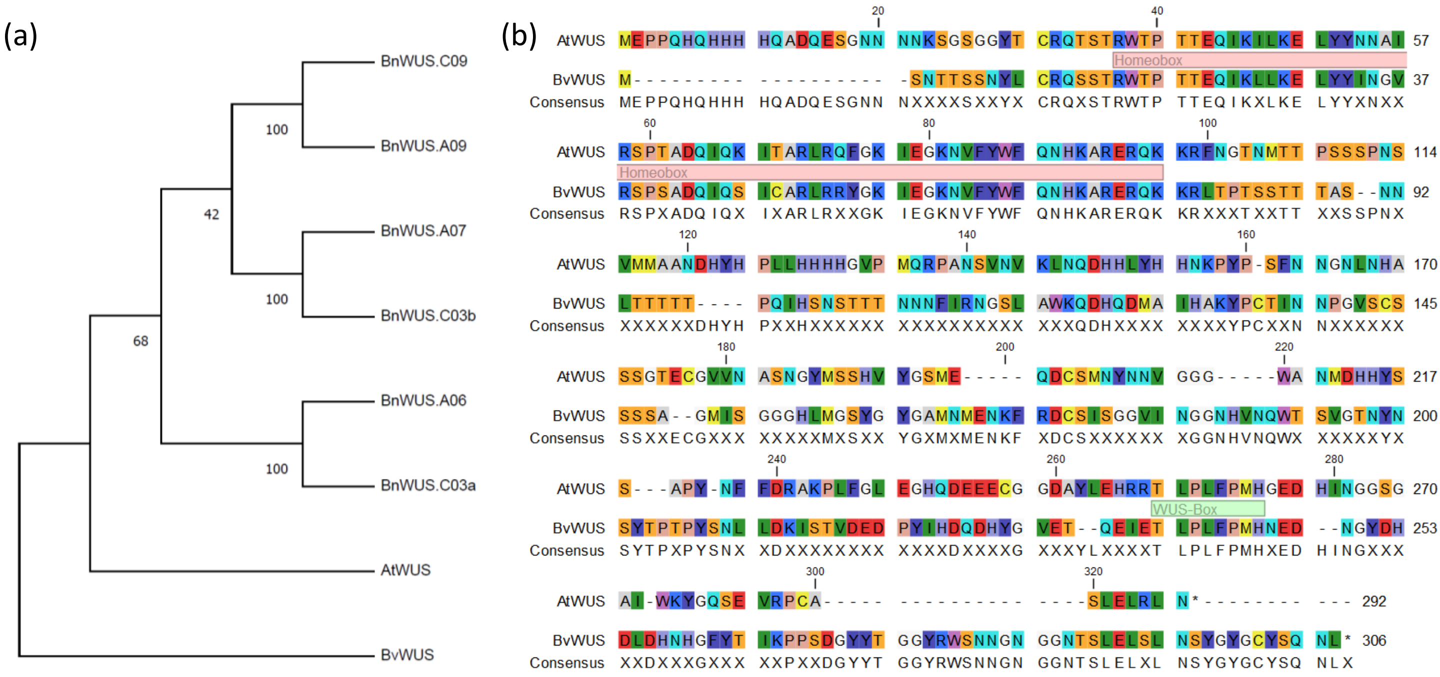

Supplementary Figure 1. (a) Evolutionary relationships of taxa. The evolutionary history was inferred using the Neighbor-Joining method (Saitou and Nei 1987). The optimal tree is shown. The percentage of replicate trees in which the associated taxa clustered together in the bootstrap test (500 replicates) are shown below the branches (Felsenstein 1985). The evolutionary distances were computed using the Poisson correction method (Zuckerkandl and Pauling 1965) and are in the units of the number of amino acid substitutions per site. This analysis involved 8 amino acid sequences. All ambiguous positions were removed for each sequence pair (pairwise deletion option). There were a total of 354 positions in the final dataset. Evolutionary analyses were conducted in MEGA11 (Tamura et al. 2021). **(b)** Amino acid alignment of AtWUS and BvWUS showing the conserved WUS-box and the variable rest of the proteins.

Felsenstein, J. (1985). Confidence Limits on Phylogenies: An Approach Using the Bootstrap. *Evolution* 39, 783–791. doi: 10.2307/2408678

Saitou, N., and Nei, M. (1987). The neighbor-joining method: a new method for reconstructing phylogenetic trees. *Molecular Biology and Evolution* 4, 406–425. doi: 10.1093/oxfordjournals.molbev.a040454

Tamura, K., Stecher, G., and Kumar, S. (2021). MEGA11: Molecular Evolutionary Genetics Analysis Version 11. *Molecular Biology and Evolution* 38, 3022–3027. doi: 10.1093/molbev/msab120

Zuckerkandl, E., and Pauling, L. (1965). “Evolutionary Divergence and Convergence in Proteins,” in *Evolving Genes and Proteins*, eds. V. Bryson and H. J. Vogel (Academic Press), 97–166. doi: 10.1016/B978-1-4832-2734-4.50017-6

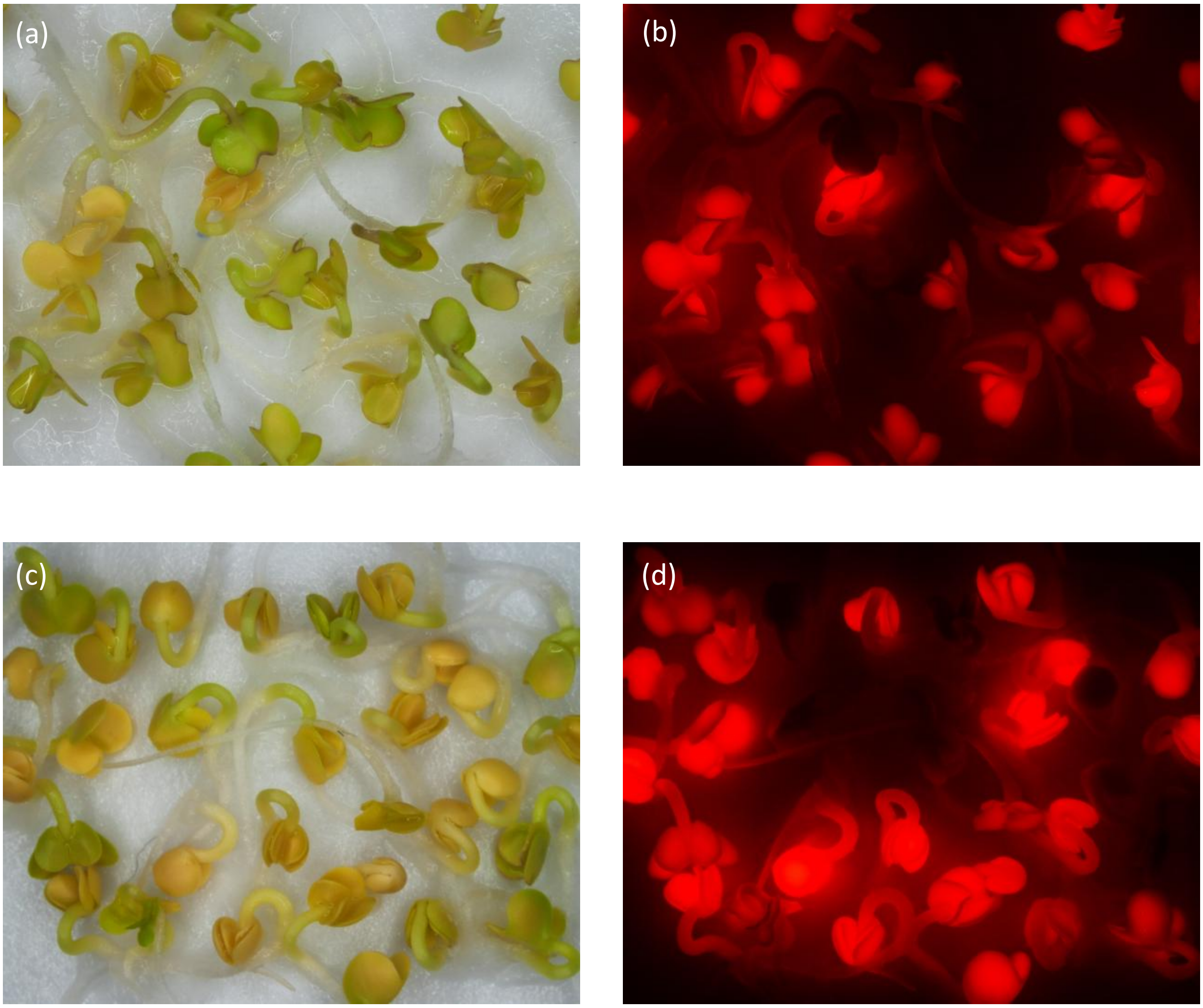

Supplementary Figure 2. Express617 *clv3-E8* T_1 seedlings in (a) white light and (b) exhibiting red fluorescence from the FAST marker. Westar *clv3-W10* T_1 seedlings in (a) white light and (b) exhibiting red fluorescence from the FAST marker.

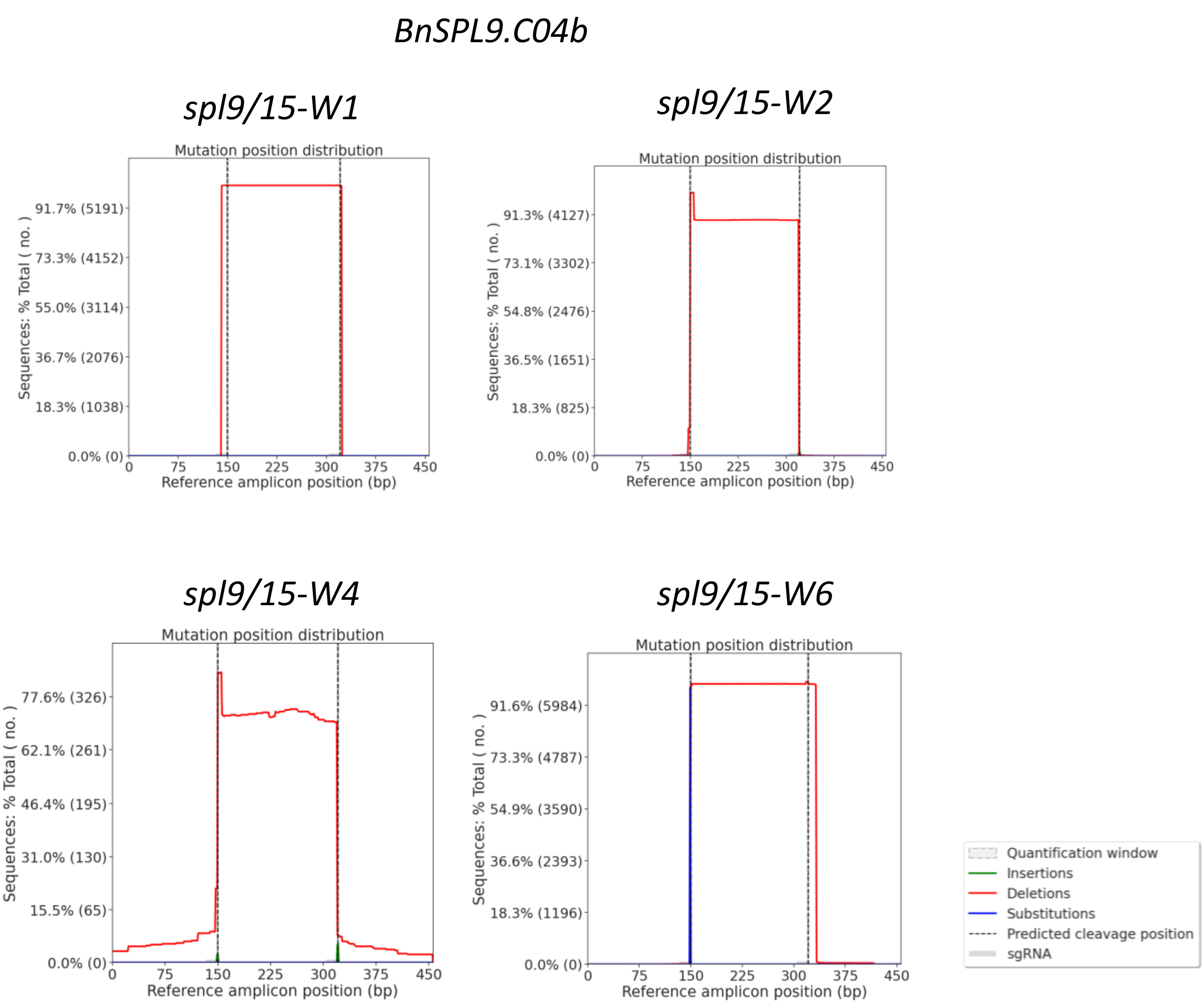

Supplementary Figure 3. CRISPResso2 results for *BnSPL9.C04b* in T_0 plants *spl9/15-W1*, *spl9/15-W2*, *spl9/15-W4*, and *spl9/15-W6*. Frequency of insertions, deletions, and substitutions across the entire amplicon, considering only modifications that overlap with the quantification window. In all four analyzed plants, deletions between the two target sites occurred.

BnSPL9.C04a

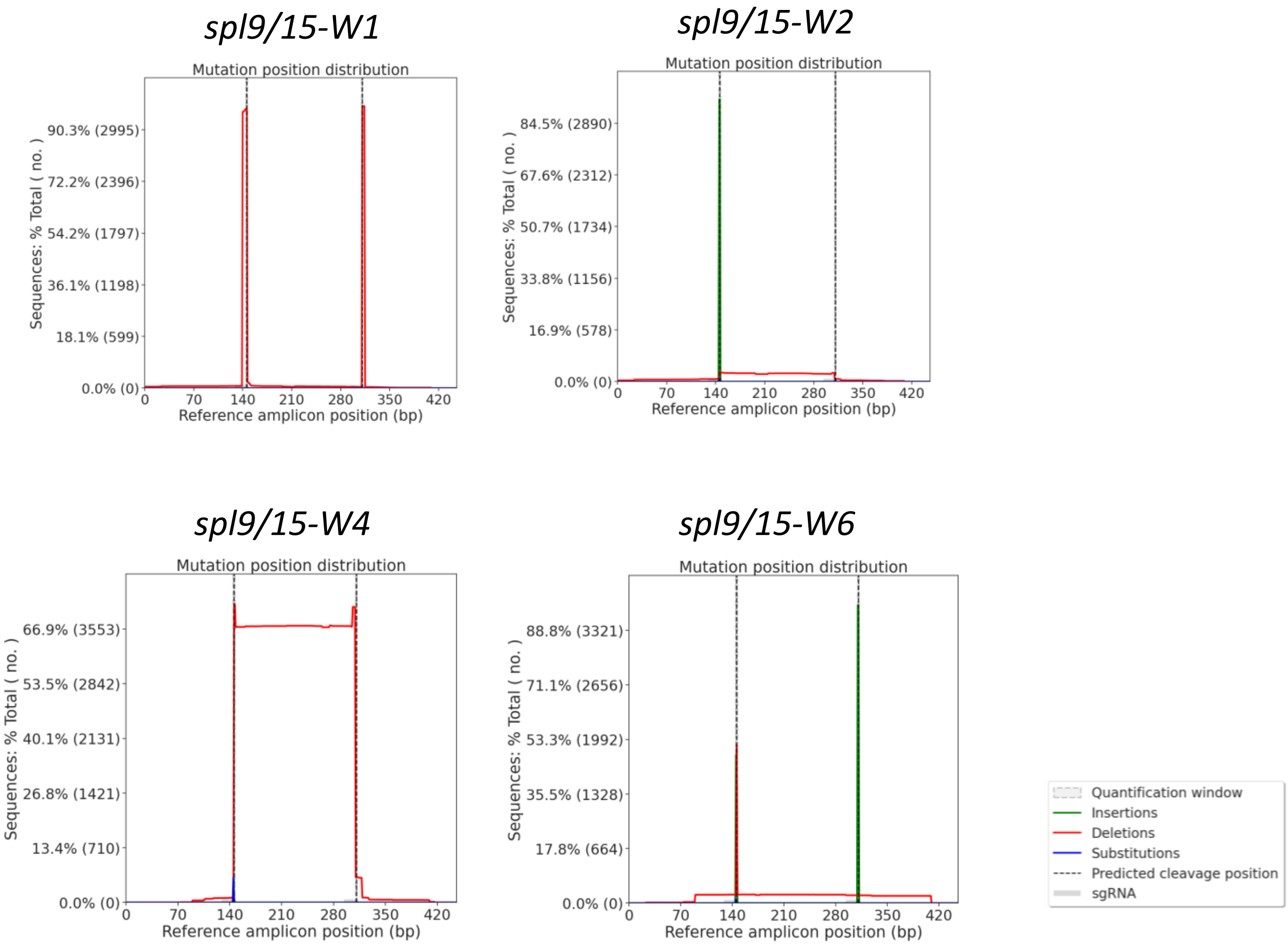

Supplementary Figure 4. CRISPResso2 results for *BnSPL9.C04a* in T_0 plants *spl9/15-W1*, *spl9/15-W2*, *spl9/15-W4*, and *spl9/15-W6*. Frequency of insertions, deletions, and substitutions across the entire amplicon, considering only modifications that overlap with the quantification window. In all four analyzed plants, deletions between the two target sites occurred.

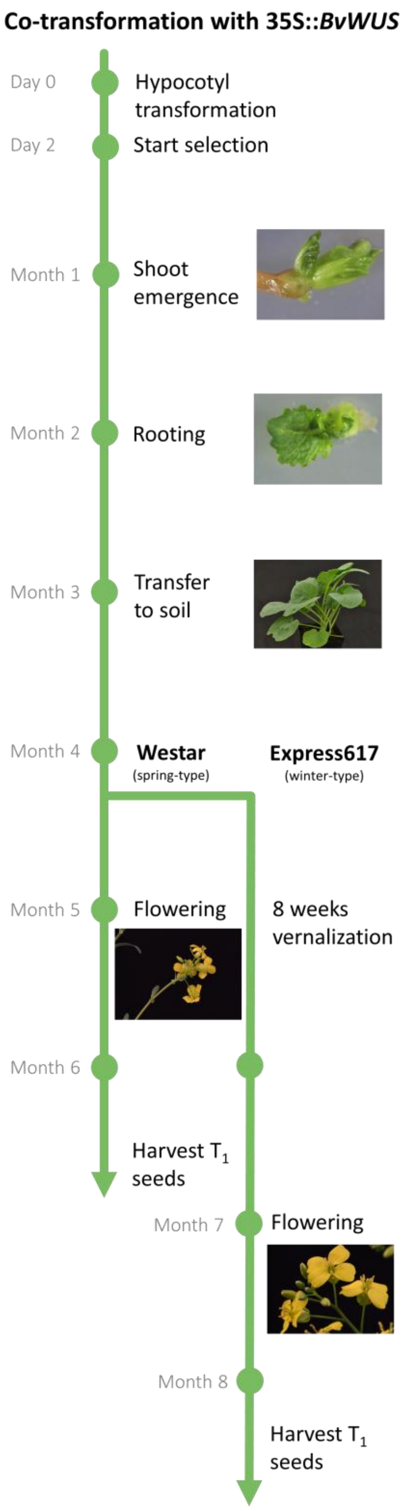

Supplementary Figure 5. Overview and timeline of the *BvWUS* co-transformation procedure.

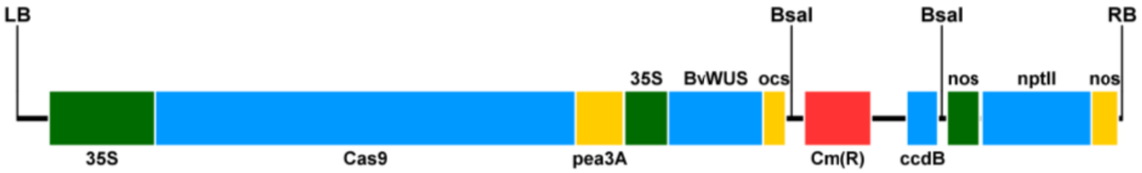

Supplementary Figure 6. T-DNA of the CRISPR vector pCas9-WUS.
