# Supplementary material for: Efficient and versatile rapeseed transformation for new breeding technologies
Source: Plant J. 2025 Jul 10;123(1):e70330. doi: 10.1111/tpj.70330 (PMC12245476; doi:10.1111/tpj.70330)
Supplement: Supplementary file 2 — Table S1. Target sites for BnCLV3 and BnSPL9/15. Table S2. Gene editing in T0 Westar and Express617 clv3 mutants. Table S3. Integration of transgenes in T1 progeny. Table S4. Gene editing in two T0 Westar spl9/15 mutants. Table S5. Gene editing in T1 Westar progeny of spl9/15‐W2 and spl9/15‐W6. Table S6. Primers used for the construction of the vector pCas9‐WUS. Table S7. Gene editing in T0 clv3 Express617 plants transformed with the pCAS9‐WUS vector. Table S8. Primers used in this study. Table S9. Gene information. [file TPJ-123-0-s001.pdf]

Supplementary Table 1: Target sites for *BnCLV3* and *BnSPL9/15*

Target gene	sgRNA	Target sequence	PAM	Oligos for sgRNA construction
BnCLV3.A04	TS1	CTCTCTACAAAATGGATTCG	AGG	attgCTCTCTACAAAATGGATTCG
BnCLV3.C04	TS1	CTCTCTACAAAATGGATTCG	AGG	aaacCGAATCCATTTTGTAGAGAG
BnCLV3.C04	TS2	ATGTTCCTGCATGGTGCTTC	TGG	attgATGTTCCTGCATGGTGCTTC
				aaacGAAGCACCATGCAGGAACAT
BnCLV3.A04	TS3	ATGTTCCTGCATGATGCTTC	TGG	attgATGTTCCTGCATGATGCTTC
				aaacGAAGCATCATGCAGGAACAT
BnCLV3.A04	TS4	TCATGCACTTCCCATTCGCA	AGG	attgTCATGCACTTCCCATTCGCA
BnCLV3.C04	TS4	TCATGCACTTCCCATTCGCA	AGG	aaacTGCGAATGGGAAGTGCATGA
BnSPL15.A07	TS1	CAGAACCAGCCCGAGTCGGC	TGG	attgCAGAACCAGCCCGAGTCGGC
BnSPL15.C06	TS1	CAGAACCAGCCCGAGTCGGC	TGG	aaacGCCGACTCGGGCTGGTTCTG
BnSPL9.A05	TS2	CCAGGCCAGACAGAGTCGGG	TGG	attgCCAGGCCAGACAGAGTCGGG
BnSPL9.C04b	TS2	CCAGGCCAGACAGAGTCGGG	TGG	aaacCCCGACTCTGTCTGGCCTGG
BnSPL9.A04	TS3	CCGGGTCAGGCAGAGTCCGG	TGG	attgCCGGGTCAGGCAGAGTCCGG
BnSPL9.C04a	TS3	CCTGGTCAGGCAGAGTCCGG	TGG	aaacCCGGACTCTGCCTGACCCGG
BnSPL15.A07	TS4	ACAGCTAGGTGCCAAGTGGA	AGG	attgACAGCTAGGTGCCAAGTGGA
BnSPL15.C06	TS4	ACAGCTAGGTGCCAAGTGGA	AGG	aaacTCCACTTGGCACCTAGCTGT
BnSPL15.A04	TS4	GGAGCTAGGTGCCAAGTGGA	AGG	
BnSPL15.C04	TS4	GCAGCTAGGTGCCAAGTGGA	AGG	
BnSPL9.C04b	TS5	ATACCAAGATGTCAAGTGGA	AGG	attgATACCAAGATGTCAAGTGGA
BnSPL9.A05	TS5	ATACCAAGGTGTCAAGTGGA	AGG	aaacTCCACTTGACATCTTGGTAT
BnSPL9.A04	TS6	ATACCAAGGTGCCAAGTGGA	AGG	attgATACCAAGGTGCCAAGTGGA
BnSPL9.C04a	TS6	ATACCAAGGTGCCAAGTGGA	AGG	aaacTCCACTTGGCACCTTGGTAT

Supplementary Table 2: Gene editing in T_0 Westar and Express617 *clv3* mutants. Mutations at the target sites are indicated by + for an insertion, - for a deletion, or are specified by more complex mutations. Deletions and insertions that are larger than 1 bp are given in bp.

**T_0 Plant**	**Allele**	**TS1**	**TS2/3**	**TS4**
clv3-W2	A04.1	+A	WT	WT
	A04.2		-185	
	C04.1	-5	WT	+A
	C04.2	+A	WT	WT
clv3-W7	A04.1	+A	- 133 bp	
	A04.2		- 54	WT
	C04.1	+G	- 125	
	C04.2	- 11	+A	+A
clv3-W8	A04.1	- 11	WT	WT
	A04.2	+A	WT	WT
	C04.1	-7	+T	WT
	C04.2	inv	WT	WT
clv3-W10	A04.1	WT	WT	WT
	A04.2	WT	WT	+A
	C04.1	WT	WT	WT
	C04.2	+A	WT	+G
clv3-E2	A04.1		- 185	
	A04.2		- 185	
	C04.1		- 53	+G
	C04.2		- 53	+G
clv3-E4	A04.1		- 185	
	A04.2		- 185	
	C04.1		- 53	+C
	C04.2		- 53	+C
clv3-E7	A04.1	+T	+T	+A
	A04.2	WT	- 125	
	C04.1	WT	WT	WT
	C04.2	+A	WT	+T
clv3-E8	A04.1	+A	WT	-1
	A04.2	-12	+A	+A
	C04.1		-43	+A
	C04.2	+C	+A	WT
clv3-E9	A04.1	+A	WT	WT
	A04.2	WT	+A	WT
	C04.1	WT	+A	WT
	C04.2	+A	+T	WT
clv3-E12	A04.1	WT	WT	WT
	A04.2	WT	-4	WT
	C04.1	WT	+A	WT
	C04.2	WT	WT	WT

Supplementary Table 3: 48 T_1 progeny per T_0 *clv3* Westar mutant were analyzed for the presence of the *BvWUS* and CRISPR transgene. Shown are the number of plants that had the respective transgene.

**T_1 Progeny**	***WUS* +**	**CRISPR +**
clv3-W2	48	48
clv3-W7	39	48
clv3-W8	0	45
clv3-W10	0	31

Supplementary Table 4: Gene editing in two T_0 Westar *spl9/15* mutants

T_0 Plant	Gene	Allele	Target site	
			**TS2**	**TS6**
spl9/15-W2	*SPL9*	A04.1	+T	WT
		A04.2	+G	WT
			**TS2**	**TS5**
		A05.1	+A	WT
		A05.2	+C	WT
			**TS3**	**TS6**
		C04a.1	+T	WT
		C04a.2	+T	WT
			**TS2**	**TS5**
		C04b.1	-170	
		C04b.2	-9	WT
			**TS1**	**TS4**
	SPL15	A07.1	+T	-2
		A07.2	+A	+T
			**TS1**	**TS4**
		C06.1	+T	+T
		C06.2	+T	+T
				**TS4**
		A04.1		+T
		A04.2		-3
				**TS4**
		C04.1		+A
		C04.2		+T

T_0 Plant	Gene	Allele	Target site	
			**TS2**	**TS6**
spl9/15-W6	*SPL9*	A04.1	+T	+T
		A04.2	+A	+T
			**TS2**	**TS5**
		A05.1	+G	+A
		A05.2	+T	+T
			**TS3**	**TS6**
		C04a.1	-C	+T
		C04a.2	+A	+T
			**TS2**	**TS5**
		C04b.1	+T	-183
		C04b.2	-G	-3
			**TS1**	**TS4**
	SPL15	A07.1	-157	
		A07.2	WT	+T
			**TS1**	**TS4**
		C06.1	+T	+T
		C06.2	+T	+T
				**TS4**
		A04.1		WT
		A04.2		+T
				**TS4**
		C04.1		-4
		C04.2		+T

Supplementary Table 5: Gene editing in T_1 Westar progeny of *spl9/15-W2* and *spl9/15-W6.*

T_0 Plant	Gene	Allele	Target site	
			**TS2**	**TS6**
spl9/15-W2.13	*SPL9*	A04.1	+T	WT
		A04.2	+G	WT
			**TS2**	**TS5**
		A05.1	+A	WT
		A05.2	+A	WT
			**TS3**	**TS6**
		C04a.1	+T	WT
		C04a.2	+T	WT
			**TS2**	**TS5**
		C04b.1	-170	
		C04b.2	-9	WT
			**TS1**	**TS4**
	SPL15	A07.1	+A	+T
		A07.2	+A	+T
			**TS1**	**TS4**
		C06.1	+T	+T
		C06.2	+T	+T
				**TS4**
		A04.1		+T
		A04.2		-3
				**TS4**
		C04.1		+T
		C04.2		+T

T_0 Plant	Gene	Allele	Target site	
			**TS2**	**TS6**
spl9/15-W2.26	*SPL9*	A04.1	+T	WT
		A04.2	+G	WT
			**TS2**	**TS5**
		A05.1	+C	WT
		A05.2	+C	WT
			**TS3**	**TS6**
		C04a.1	+T	WT
		C04a.2	+T	WT
			**TS2**	**TS5**
		C04b.1	-170	
		C04b.2	-9	WT
			**TS1**	**TS4**
	SPL15	A07.1	+T	-2
		A07.2	+A	+T
			**TS1**	**TS4**
		C06.1	+T	+T
		C06.2	+T	+T
				**TS4**
		A04.1		+T
		A04.2		+T
				**TS4**
		C04.1		+T
		C04.2		+T

T_0 Plant	Gene	Allele	Target site	
			**TS2**	**TS6**
spl9/15-W6.21	*SPL9*	A04.1	+T	+T
		A04.2	+A	+T
			**TS2**	**TS5**
		A05.1	+G	+A
		A05.2	+T	+T
			**TS3**	**TS6**
		C04a.1	-C	+T
		C04a.2	-C	+T
			**TS2**	**TS5**
		C04b.1	+T -183	
		C04b.2	+T -183	
			**TS1**	**TS4**
	SPL15	A07.1	-157	
		A07.2	WT	+T
			**TS1**	**TS4**
		C06.1	+T	+T
		C06.2	+T	+T
				**TS4**
		A04.1		+T
		A04.2		+T
				**TS4**
		C04.1		+T
		C04.2		+T

T_0 Plant	Gene	Allele	Target site	
			**TS2**	**TS6**
spl9/15-W6.32	*SPL9*	A04.1	+A	+T
		A04.2	+A	+T
			**TS2**	**TS5**
		A05.1	+G	+A
		A05.2	+T	+T
			**TS3**	**TS6**
		C04a.1	-C	+T
		C04a.2	+A	+T
			**TS2**	**TS5**
		C04b.1	+T -183	
		C04b.2	+T -183	
			**TS1**	**TS4**
	SPL15	A07.1	-157	
		A07.2	WT	+T
			**TS1**	**TS4**
		C06.1	+T	+T
		C06.2	+T	+T
				**TS4**
		A04.1		+T
		A04.2		+T
				**TS4**
		C04.1		+T
		C04.2		+T

Supplementary Table 6: Primers used for the construction of the vector pCas9-WUS. Small letters indicate the overhangs of primers with restriction sites.

Primer	Sequence 5'–3'
Mut1_BsaI_for	CTCTTTCCTGTGGATAGC
Mut1_BsaI_rev	CCTTTTCGACCTTTTTCC
Mut2_BsaI_for	TCTCATCAAAGACGATCTACCCGAG
Mut2_BsaI_rev	CTGCCGCGTAGGCCTCTC
HindIII_Kan_for	agttaagcttAGCGGAGAATTAAGGGAGTC
HindIII_Kan_rev	attcaagcttTCGATCTAGTAACATAGATGACACCG
XbaI_WUS_for	tcgatctagaTCAACATGGTGGAGCACG
XbaI_WUS_rev	ggcatctagaGGACAATCAGTAAATTGAACGGAG
XbaI_ccdb_for	tcgatctagaTTGCAGAGACCGAGACGTT
XbaI_ccdb_rev	ggcatctagaAAGCTGAGACCGACTGGCT

Supplementary Table 7: Mutations in *BnCLV3* in Express617 plants transformed with the pCAS9-WUS vector.
Mutations at the target sites are indicated by + for an insertion, ´ for a deletion. Deletions and insertions that are larger than 1 bp are given in bp. Substitutions are indicted by ->.

**Name**	***clv3* phenotype**	**Allele**	**TS1**	**TS2/3**	**TS4**
clv3-E1-pCAS9	-	A04.1	-4 (ATG)	WT	WT
		A04.2	T -> G	WT	WT
		C04.1	+T	+A	WT
		C04.2	-5 (ATG)	WT	WT
clv3-E2-pCAS9	+	A04.1	-9	+T	WT
		A04.2	-6	WT	+C
		C04.1	-6 (ATG)	+T	WT
		C04.2	-3	+C	WT
clv3-E3-pCAS9	-	A04.1	-6 (ATG)	WT	WT
		A04.2	-6 (ATG)	WT	WT
		C04.1	+T	WT	WT
		C04.2	WT	WT	WT

Supplementary Table 8: Primers used in this study.

Primer	Sequence
AtWUS_BamHI_for	AGTTGGATCCATGGAGCCGCCACAGCATCA
AtWUS_EcoRI_rev	ATTCGAATTCTAGTTCAGACGTAGCTCAA
BvWUS_BamHI_for	AGTTGGATCCATGAGTAATACTACAAGTAG
BvWUS_EcoRI_rev	ATTCGAATTCCTAAAGATTTTGTGAATAAC
AtWUS_for	AGCCGATCAGATCCAGAAGA
AtWUS_rev	AACCGAGTTGGGTGATGAAG
BvWUS_for	TTCCTCTCTTCCCAATGCAC
BvWUS_rev	AGGGAGGTATTCCCACCATT
Kan1-r	CTTCCCGCTTCAGTGACAAC
Kan2-f	TTGGGTGGAGAGGCTATTCG
CLV3_A04_for	GTGTTCTATATTCCGGACATACG
CLV3_A04_rev	CTGAAGGGACAGTCCTTAGT
CLV3_C04_for	GGAGAAAGGATCTAGTGATCG
CLV3_C04_rev	GCTAAGGACTGTCCCTTCAG
NGS_CLV3_for	CTTGCAGCCTATAAATGATTGC
NGS_CLV3_rev	AACACGAGATAGATGTCCG
SPL9_A04_for	CACAGTTGGTTGATAAGCATTTAG
SPL9_A04_rev	CAGACCGTGTTAGCTTCTAGA
SPL9_A05_for	CATGAACCAAGCGATGAGTAC
SPL9_A05_rev	CCTGGTCCCATAACATTATGC
SPL9_C04a_for	GCTGGAACTGCCTTATGTTG
SPL9_C04a_rev	CAGTCCGTGTTAACTTCTAAGG
SPL9_C04b_for	GCAAGTACTTAAAGGTCGTAACC
SPL9_C04b_rev	ACCATTTCCTGGCCCATG
SPL15_A07_for	AGATGTTCACTACAGAAAACG
SPL15_A07_rev	ACCTAACCATATAGAGATGGAGAG
SPL15_C06_for	GTTCATTCACTGCAGAGAACC
SPL15_C06_rev	CATATAGAGATGGAGCGATGTTG
NGS_SPL9_A04_for	CGTCCTTTCTTTAAACCAAGACAG
NGS_SPL9_A05_for	CCTTTCCTTTAAACCGAGACAG
NGS_SPL9_C04a_for	GTCCTTTCTTCAAACCAAGACAG
NGS_SPL9_C04b_for	CCTTGCCTTTAAACCGAGACAG
NGS_SPL9_rev	GAACCTGCTGCACTGTTGAC
NGS_SPL15A04_C04_for	TGGAGTTACTAATGGGTTCGG
NGS_SPL15A04_C04_rev	CATTGTTGGCAAAACCTTTGA
NGS_SPL15A07_C06_for	TCGGCTGGTTCCTCGTCTA
NGS_SPL15A07_C06_rev	CATTGCTGGCAAAACCTTTGG

Supplementary Table 9: Gene information.

Species	Genotype	Gene	Gene identifier	Reference genome
B.napus	Express617	BnCLV3	A04p016980.1_BnaEXP	Express617 (Lee et al., 2020)
			C04p038910.1_BnaEXP	
B.napus	Westar	BnCLV3	BnaA04T0166900WE	Westar v0 (Song et al., 2020)
			BnaC04T0465000WE	
		BnSPL9	BnaA04T0257100WE	
			BnaC04T0572100WE	
			BnaC04T0031400WE	
			BnaA05T0019400WE	
		BnSPL15	BnaA04T0025800WE	
			BnaC04T0286900WE	
			BnaC06T0236300WE	
			BnaA07T0194600WE	

References

Lee, H., Chawla, H. S., Obermeier, C., Dreyer, F., Abbadi, A., and Snowdon, R. (2020). Chromosome-Scale Assembly of Winter Oilseed Rape *Brassica napus*. *Frontiers in Plant Science* 11. doi: 10.3389/fpls.2020.00496

Song, J.-M., Guan, Z., Hu, J., Guo, C., Yang, Z., Wang, S., et al. (2020). Eight high-quality genomes reveal pan-genome architecture and ecotype differentiation of *Brassica napus*. *Nature Plants* 6, 34–45. doi: 10.1038/s41477-019-0577-7
